# Geospatial Information Research: State of the Art, Case Studies and Future Perspectives

**DOI:** 10.1007/s41064-022-00217-9

**Published:** 2022-09-19

**Authors:** Ralf Bill, Jörg Blankenbach, Martin Breunig, Jan-Henrik Haunert, Christian Heipke, Stefan Herle, Hans-Gerd Maas, Helmut Mayer, Liqui Meng, Franz Rottensteiner, Jochen Schiewe, Monika Sester, Uwe Sörgel, Martin Werner

**Affiliations:** 1 Geodäsie und Geoinformatik, Universität Rostock, Rostock, Germany; 2 Geodätisches Institut der RWTH Aachen, Aachen, Germany; 3 Lehrstuhl Geoinformatik, KIT, Karlsruhe, Germany; 4 Geoinformation, Institut für Geodäsie und Geoinformation, Universität Bonn, Bonn, Germany; 5 Institut für Photogrammetrie und GeoInformation, Leibniz Universität Hannover, Hannover, Germany; 6 Photogrammetrie, TU Dresden, Dresden, Germany; 7 Visual Computing, Universität der Bundeswehr München, Neubiberg, Germany; 8 Lehrstuhl für Kartographie und Visuelle Analytik, Technische Universität München, Munich, Germany; 9 Geoinformatik and Geovisualisierung, HafenCity Universität Hamburg, Hamburg, Germany; 10 Institut für Kartographie und Geoinformatik, Leibniz Universität Hannover, Hannover, Germany; 11 Institut für Photogrammetrie, Universität Stuttgart, Stuttgart, Germany; 12 Big Geospatial Data Management, Technische Universität München, Munich, Germany

**Keywords:** Geospatial information sciences, Geographic information systems (GIS), Research perspectives, Grand challenges, Geoinformationswissenschaft, Geo-Informationssysteme (GIS), Forschungsperspektiven, Große gesellschaftliche Herausforderungen

## Abstract

Geospatial information science (GI science) is concerned with the development and application of geodetic and information science methods for modeling, acquiring, sharing, managing, exploring, analyzing, synthesizing, visualizing, and evaluating data on spatio-temporal phenomena related to the Earth. As an interdisciplinary scientific discipline, it focuses on developing and adapting information technologies to understand processes on the Earth and human-place interactions, to detect and predict trends and patterns in the observed data, and to support decision making. The authors – members of DGK, the Geoinformatics division, as part of the Committee on Geodesy of the Bavarian Academy of Sciences and Humanities, representing geodetic research and university teaching in Germany – have prepared this paper as a means to point out future research questions and directions in geospatial information science. For the different facets of geospatial information science, the state of art is presented and underlined with mostly own case studies. The paper thus illustrates which contributions the German GI community makes and which research perspectives arise in geospatial information science. The paper further demonstrates that GI science, with its expertise in data acquisition and interpretation, information modeling and management, integration, decision support, visualization, and dissemination, can help solve many of the grand challenges facing society today and in the future.

## Abstract (de)

*Geoinformationsforschung: Stand der Technik, Fallstudien und Zukunftsperspektiven.* Die Geoinformationswissenschaft (GI-Wissenschaft) befasst sich mit der Entwicklung und Anwendung geodätischer und informationswissenschaftlicher Methoden zur Modellierung, Erfassung, gemeinsamen Nutzung, Verwaltung, Erkundung, Analyse, Synthese, Visualisierung und Bewertung von Daten über raum-zeitliche Phänomene im Zusammenhang mit der Erde. Als interdisziplinäre wissenschaftliche Disziplin konzentriert sie sich auf die Entwicklung und Anpassung von Informationstechnologien, um Prozesse auf der Erde und Interaktionen zwischen Mensch und Raum zu verstehen, Trends und Muster in den beobachteten Daten zu erkennen und vorherzusagen sowie die Entscheidungsfindung zu unterstützen. Die Autoren – Mitglieder der Abteilung Geoinformatik im Ausschuss für Geodäsie der Bayerischen Akademie der Wissenschaften, die die geodätische Forschung und die universitäre Lehre in Deutschland vertreten – haben diesen Beitrag erstellt, um zukünftige Forschungsfragen und -richtungen in der Geoinformationswissenschaft aufzuzeigen. Für die verschiedenen Facetten der GI-Wissenschaft wird der Stand der Technik dargestellt und mit meist eigenen Fallbeispielen untermauert. Der Aufsatz verdeutlicht damit, welche Beiträge die deutsche GI-Community leistet und welche Forschungsperspektiven sich in der GI-Wissenschaft ergeben. Darüber hinaus wird gezeigt, dass die GI-Wissenschaft mit ihrer Expertise in der Datenerfassung und -interpretation, der Informationsmodellierung und dem Informationsmanagement zur Lösung vieler der großen gesellschaftlichen Herausforderungen von heute und morgen beitragen kann.

## Introduction and Motivation

Geospatial information science (in German mostly called Geoinformatik) is concerned with the development and application of geodetic and computer science methods for the modeling and acquisition, exchange, management, exploration, analysis, synthesis, visualization, and evaluation of data concerning space–time-variant phenomena related to the Earth. As a scientific discipline, it focuses on developing and adapting information technologies to understand places and processes on the Earth, to support human behavior and decision making, and to detect and predict trends and patterns in the observed data. Geospatial information technologies contribute to the mapping and analysis of the Earth and the grand challenges facing human societies such as climate change, demographic change, sustainable agriculture and forestry, environmental degradation, energy security, resource efficiency, mobility.

Geospatial information processing integrates research issues and information products of many scientific disciplines, such as photogrammetry, remote sensing, cartography, engineering geodesy, and spatial observations in geography and environmental sciences, and transforms them into structured information as well as into maps and other communication media suitable for humans. To solve these tasks, besides concepts from mathematics and physics, various subareas of computer science are adapted and extended in an engineering-oriented manner, such as computational geometry, artificial intelligence and machine learning, semantic technologies, databases, and computer graphics (see also Kutterer et al. 2020).

Advances in sensor and positioning technologies in recent years have facilitated unprecedented growth in the collection of spatially and temporally referenced data. Classical examples of big spatial data sources include aerial and terrestrial laser scanning, remote sensing imagery, and weather data. In addition, gigantic amounts of (in many cases low-cost miniaturized) sensors generate real-time data streams in everyday life in the context of the Internet of Things (IoT) (for geospatial-IoT see Sect. 8). Millions of people voluntarily contribute to the collection of geoinformation (for example in OpenStreetMap (OSM) or in citizen science projects) or share posts with a spatial reference in the social networks and thus create so-called volunteered geographic information (VGI). Tracks of various objects moving by land, sea, and air will become more and more available in the context of digital earth, smart cities, humans as sensors, citizen science, location-based services (LBS),”in-situ” geocomputing, social media, etc. VGI data encompasses many specific types of data, such as geotagged social media (Twitter, Instagram, etc.), geotagged Wikipedia pages, news articles, historical archives, location-focused online reviews, geotagged housing posts, and others that contain links between locations and interpretable information.

Location is always an available and important common property of all of these new types of information. As Goodchild (2009) already stated: “it will be possible to know where everything is, at all times.” This will result in new research topics and challenges, as Andrienko et al. (2017) predicted: “The massive volumes of collected data contain complex, yet implicit spatial, temporal, and semantic interrelations that are waiting to be uncovered and made explicit.” Craglia et al. (2012) described the concept of the “Observation Web” with observations originating from humans, sensors or numerical (environmental) simulations, and others, moving from an essentially static representation of the Earth to one that is dynamic and interactive, and more responsive to the grand challenges (see Sect. 10).

With the advent of the IoT and big data, citizens have increasingly been involved in producing and using a new type of information useful for analyzing spatial problems, and researchers and practitioners increasingly have been confronted with the task of developing methods and approaches for massive data collection, integration, and analysis in spatially explicit formats (Malczewski and Jankowski 2020). The fundamental (i.e. special) properties of spatial data—spatial dependence, spatial heterogeneity, and concepts such as location, distance, direction, connectivity, adjacency, neighborhood, proximity—need to be rethought considering IoT and big data issues. New solutions for data storage and data processing, for example for semi-structured data, or technologies for parallelization and distributed computing are gaining importance. The problem of semantic heterogeneity caused by different meanings of data, terminologies, and models needs to be solved. The analysis of these heterogeneous data and the preparation of decision-supporting statements represent an important task for geoinformatics (Kutterer et al. 2020). The investigation of methods to validate these huge amounts of data against each other as well as against official authoritative data, knowledge bases, and simulation results is necessary. The integration of these geo-observation webs with common earth observation infrastructures, and the dynamic and interactive, as well as automatic exploitation of spatio-temporal mass data variety and flows from sensors and people is a future challenge.

In the context of mobility, data from permanently measuring sensors (e.g. floating car data) plays an increasingly important role alongside 3D city models. In smart cities, measures are to be derived and implemented automatically from sensor data and social and behavioral implications need to be taken into account (see also Huang et al. 2018).

The Committee on Geodesy of the Bavarian Academy of Sciences and Humanities (formerly Deutsche Geodätische Kommission, abbreviated "DGK", https://dgk.badw.de/) represents geodetic research and university teaching in Germany. The DGK division “Geoinformatik” (https://dgk.badw.de/abteilung-geoinformatik.html) initiates and coordinates research projects, discusses future trends and scientific perspectives in geospatial information science, and maintains professional exchange. The division focuses on topics such as the acquisition of geospatial data and the derivation of digital descriptions of environmental objects at different scales and as fully automated as possible. The aim is not only to record geometric properties but also to provide descriptions of a range of different object properties (e.g. building function, terrain shapes, movement patterns, land use types, human behavior) and temporal information by automated merging and integration of geodata from different sources. The development of common data models to create (semantic) reference systems and the establishment of modern geodata infrastructures based on spatial information theory is another topic of DGK. The digital geodata collections that are emerging in large numbers and growing in size with modern sensor technologies require automatic spatial analysis methods, for example, to derive heat and energy losses in construction planning, to recognize movement patterns in mobility, or to determine damages for disaster management.

Members of the DGK division “Geoinformatik” have prepared this paper to assess the current situation and to point out future research questions and directions in geospatial science, building upon previous efforts by ISPRS (International Society for Photogrammetry and Remote Sensing) (Chen et al. 2016), ICA (International Cartographic Association) (Virrantaus et al. 2009; Meng et al. 2020), and others. We identify the scientific research challenges for the next decade and describe how we will contribute our expertise along the whole information processing chain in GI technology to tackle them.

The structure of the article is as follows: Sects. 2, 3, 4, 5, 6, 7, 8 treat research aspects of GI science in information acquisition and geometric processing, information interpretation, information modeling and management, information integration, decision support, geospatial visualization, and information dissemination. Each step along this processing chain is reflected with a brief description of the status quo and underlined by a few selected case studies, mainly from the authors. Then, we look ahead to the most important research trends of the next decade. Section 9 briefly considers relevant infrastructures and research funding to support these research issues. Finally, we address the grand challenges, from the point of view of geospatial information processing, to reach a certain consensus in the scientific community about the contributions of the GI community to these major societal challenges.

## Geospatial information acquisition and geometric processing

### Developments in data acquisition and new applications

Recent developments in sensor technology have led to a tremendous increase in spatially and temporally referenced data. Examples include aerial, satellite, but also terrestrial images, 3D point clouds from laser scanners and interferometric synthetic aperture radar (InSAR), trajectory data using e.g. portable GNSS receivers, and volunteered geographic information. Sensors are mounted on satellites, airplanes, unmanned aerial vehicles (UAVs), and static as well as mobile terrestrial platforms such as mobile mapping systems. Increasingly, multisensor systems and geosensor networks are being used for cooperative data acquisition.

In this section, we give a short description of developments in geospatial data acquisition. Since our focus is on data handling and not on sensor hardware development, we only point out major trends, without going into detail, primarily to illustrate the fact that geospatial data are big data—with the related challenges of an enormous increase in volume, variety, velocity, and veracity. In addition, while we note the tremendous importance of ubiquitous location and localization (according to Goodchild, 2019 “it will be possible to know where everything is, at all times”) we will not discuss developments of how to determine sensor position for the sakes of positioning and navigation in this section, but only to georeference geospatial data captured by the sensor in question.

Over the years, many national and supranational agencies, e.g., NASA, ESA, CNES, DLR, ISRO, JAXA, CNSA, developed civil *satellite remote sensing systems*. The US Landsat program, dating back to 1972, was the first source of seamless optical Earth Observation (EO) imagery suitable for certain resource mapping purposes. Since 2014, the European Copernicus program (Sentinel fleet) provides free-of-charge multispectral imagery with a very high revisit frequency and a ground sampling distance (GSD) of up to 10 m; it will continue to do so for the next decades. Other countries such as India and China have similar programs. In addition, constellations like the Pléiades Neo satellites with a GSD of 0,3 m (the first two of which were launched in April and August 2021), and the Planet constellation with more than a hundred operational satellites in orbit with a GSD in the meter range, are currently being launched, partly by private companies. Some of these satellites can capture motion in short high-resolution videos. The meter range is also the resolution of currently established radar constellations, most of which, however, seems to target the defense market (e.g., Iceye, Umbra Lab, Capella Space). For all these constellations the main goal is a high temporal resolution. In addition to the constellations, new high-resolution optical satellites have been announced, e.g., Maxar Legion with a resolution in the range of a quarter of a meter and up to 15 images of the same location per day, which increasingly blurs the separation between satellite and aerial imagery. Novel SAR missions particularly suitable for SAR interferometry like TanDEM-L (https://www.dlr.de/hr/tdml) as well as hyperspectral sensors such as the recently launched EnMAP (https://www.enmap.org/) will produce more data.

In *aerial data acquisition*, one observes an ongoing integration of nadir-looking sensors with image sizes of up to 400 Megapixel using oblique cameras, and partly also with laser scanners leading to colored 3D point clouds. Current laser scanning systems increasingly employ full-waveform digitization or single-photon counting techniques (Mandlburger et al. 2019), enabling them, for instance, to generate vertical vegetation density profiles in forests as a basis for biomass change quantification and to capture terrain model data at a rate of several million points per second. Aerial radar for civil applications is still a niche market.

While for a long time *unmanned aerial vehicles* in most cases only carried cameras, recently, laser scanners with sufficient range have become smaller and lighter and, most importantly, have been integrated with sufficiently high-precision lightweight GNSS and INS sensors. In addition, thermal and hyperspectral sensors for UAVs are available, although the geometric resolution is still limited. Concerning the availability of suitable carrier systems, one has to note that the (semi)-professional area is growing rapidly, e.g., for taking video footage of sports events or for surveillance purposes.

For several years there has been a trend towards Computational Photography (CP, Nayar 2007) in *terrestrial data acquisition*. These cameras often have multiple lenses: images are taken with different settings and are combined computationally. An example is plenoptic cameras, also called light-field cameras, the concept of which has been investigated for some time already (Adelson et al. 1992). Computational photography is particularly being employed in recent mobile phones. High-speed cameras at frequencies, ranging up to 1 MHz and above, are used to acquire dynamic scenes in areas such as sports, traffic monitoring, surveillance, robotics, autonomous driving, crash tests, material testing, and fluid dynamics. For terrestrial sensing the same thermal and hyperspectral sensors can be used as those mounted on UAVs, meaning that the geometric resolution is rather limited. In many cases, sensors are combined into a multisensor platform with various different cameras, laser scanners, and positioning devices carried by humans or cars; mobile mapping systems traveling in normal traffic are a particular example of this development. While mobile mapping has become a standard tool for the detailed acquisition of urban areas, the scope has been considerably widened by the interest in HD (High Definition) maps for autonomous driving. In this context, the automatic acquisition of information about the surroundings of a vehicle using sensors such as (stereo-) cameras and laser scanners is of vital importance.

For moving objects, *trajectory data* (3D position and 3D rotation as a function of time) are an additional data source. They are typically captured using GNSS receivers and GNSS/IMU systems, but also using cameras or laser scanners. Trajectories can be used to analyze and predict object behavior. They can also serve as an additional information source for the interpretation of scenes in which these objects are depicted, e.g. using a camera or laser scanner.

Given all these developments in data acquisition, one can expect more detail concerning geometry, semantics, extent and time in the future. For instance, *city models* will be fully 3D and will contain window and door objects for the façades, possibly with mullions and transoms (horizontal and vertical bars), but also objects for stairs, balconies, dormers, chimneys, air conditioners, and more generally, street furniture and vegetation objects. Data from all platforms mentioned before will be fused and used in combined approaches to reach these goals.

Reconstruction for *Building Information Models* (BIMs) (see Sect. 4) goes further by considering, besides the interior of buildings, the semantics and the geometry of the parts they are constructed of, and possibly, also the construction process. Models of interior spaces will become common for shopping centers, but also for large public buildings such as railway stations, town halls, or museums. Smooth navigation from indoors to outdoors and vice-versa is a particular problem in this regard.

In addition to the more detailed geometry and semantics, scenes to be observed and models to describe these varying scenes will be more and more dynamic, opening up possibilities to also model, observe and understand *processes of all kinds*, e.g. landslides and other geomorphologic changes on the Earth surface, but also in sports or when observing public places. This pertains to the interior spaces of shopping malls but even more so to information related to traffic and other applications in which image sequences are commonly used.

*Land use* is another area where temporal dynamics are important. Utilizing the capabilities of the satellite constellations, a much higher acquisition frequency and, thus, a much more detailed analysis of the temporal dynamics is possible, also leading to higher update rates. Such results are required not only by agriculture and forestry but in particular, also for determining indicators to monitor progress in achieving the UN Sustainable Development Goals (SDG, https://sdgs.un.org/goals). Concerning land use, additional radar missions will be helpful, particularly for areas that are often clouded such as rain forests. In addition, advanced radar capabilities will be particularly useful for surveillance applications where all-weather capabilities are essential and higher geometric and temporal resolutions allow for a more detailed analysis.

An emerging field is a perception of *autonomous driving*. Besides ego-motion, the detection and pose estimation of other road users such as cars, cyclists, and pedestrians are essential for path planning and accident avoidance and support collaborative positioning of vehicles in GNSS-denied areas (Coenen and Rottensteiner 2019). It is also important to generate this information over time by analyzing time series, which involves tracking based on physical models of the movement of these objects over time, potentially considering interactions between different road users in a scene (Leal-Taixé et al. 2017).

### State of the Art and Case Studies in Geometric Processing

Geometric processing comprises calibration and synchronization of single sensors and sensor systems, the determination of sensor orientation (pose), the determination of (potentially highly accurate) 3D point coordinates, the 3D reconstruction of scenes, and object rendering including orthoprojection. In our context, we also consider motion models, both for sensor movement (e.g. when dealing with line sensors) and for temporal changes in object space (e.g., when determining scene flow), as part of geometric processing. Note that the determination of ego-motion, as a core requirement in navigation (e.g., using GNSS/IMU systems as the only sensor), is not treated in this section.

Optical 3D measurement techniques (in the past often referred to as close-range photogrammetry) have found a huge market potential in industrial measurement tasks, where they are applied in manifold design, manufacturing, and quality control processes. Herein, photogrammetry provides, among others, sensor modeling and self-calibration techniques allowing to achieve measurement accuracies beyond 1:100,000 of the object dimensions using off-the-shelf cameras. Image engineering techniques such as structured light approaches allow for real-time 3D measurement systems with high spatial and temporal resolution.

#### Case Study “3D Surface Reconstruction Using Images from the Ground and UAVs”

**Fig. 1:**
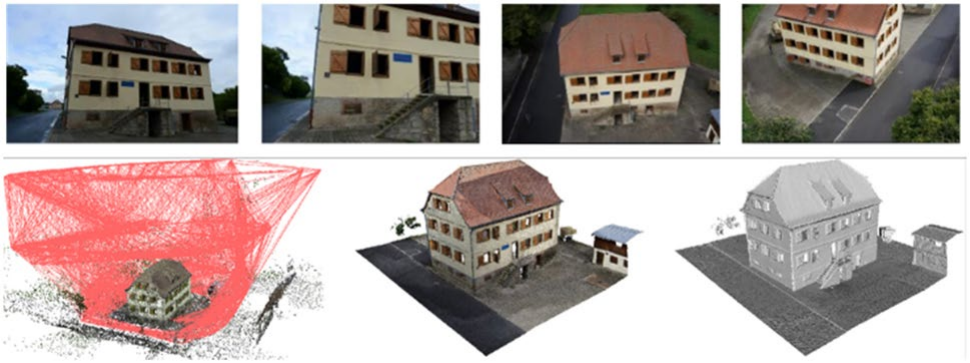
Images from the ground and from a UAV (top), orientations (projection centers are linked if the images overlap; left), and 3D model in the form of a mesh with (center) and without texture (right)

Images from different sources (see Fig. 1) such as from the ground and small UAVs allow for a detailed 3D reconstruction of the roof as well as the façades of buildings. Michelini and Mayer (2020) present an approach for the automatic orientation of unsorted images which can deal with the wide baselines occurring in this study. The orientations are the basis for pairwise image matching leading to per-pixel depth maps. These maps are combined based on their estimated accuracy using a probabilistic volumetric approach (Kuhn et al. 2017) leading to a scalable high-quality 3D reconstruction.

#### Case Study “Uncertainty Estimation of 3D Surfaces”

**Fig. 2:**
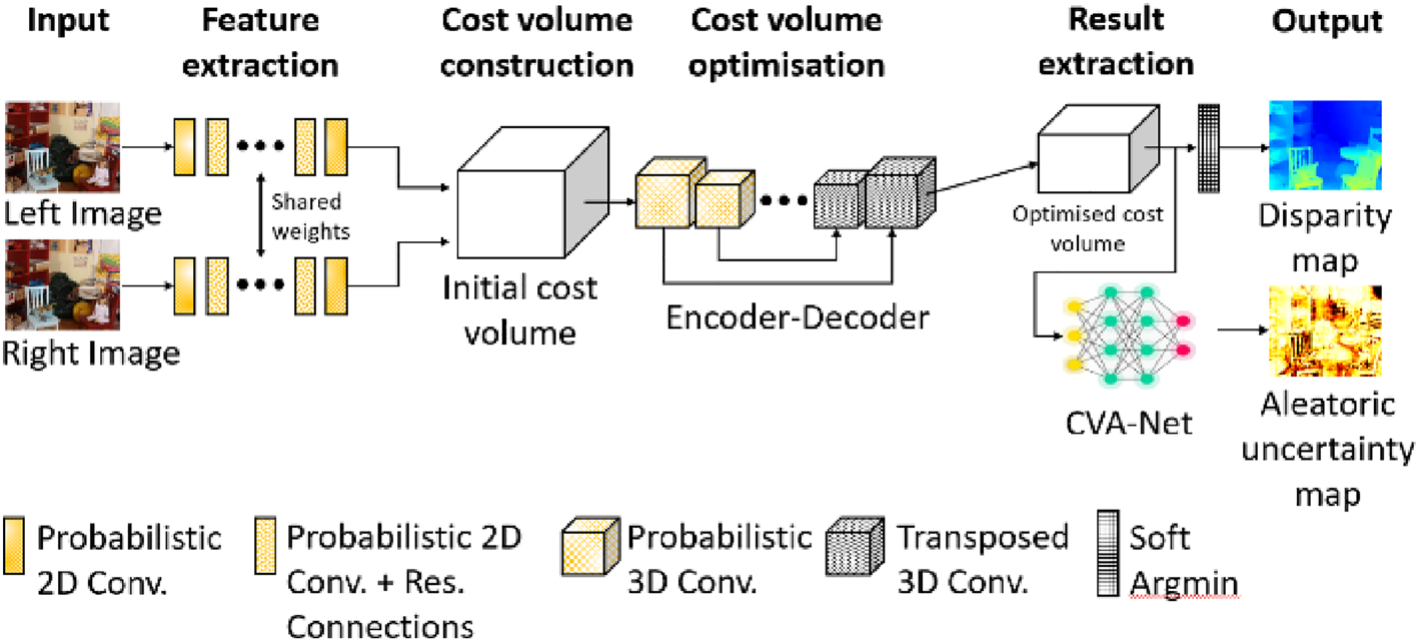
Deep learning network for the joint estimation of aleatoric and epistemic uncertainty in dense stereo matching (Mehltretter 2021)

While dense stereo matching methods have made great progress over the last few years, there is a need for self-diagnosis, i.e. to identify erroneous disparity estimates in the results. Based on probabilistic convolutional neural networks, Mehltretter (2021) presents a new method for the estimation of aleatoric and epistemic uncertainty (corresponding to stochastic and systematic uncertainty). Instead of relying on features learned from disparity maps only, the corresponding 3D cost volumes are employed. For aleatoric uncertainty estimation, a novel convolutional neural network architecture is presented that is trained with different stochastic models that follow the concept of Bayesian deep learning. The quantification of epistemic uncertainty is realized using a Bayesian neural network trained with variational inference. Figure 2 shows the design of the employed network. The results demonstrate that the models used to estimate aleatoric uncertainty outperform state-of-the-art methods. Moreover, the usage of a Bayesian neural network not only allows for epistemic uncertainty estimation but also supports the task of dense stereo matching itself, reducing the number of errors contained in the disparity maps.

#### Case Study “Material Testing”

**Fig. 3:**
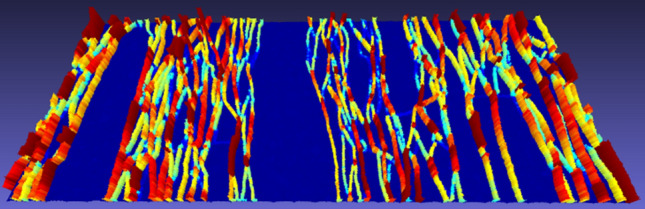
Crack pattern of a strain-hardening cement-based composite probe in a tension test, visualized by color-coding and height of the prism cells for better visual interpretability (Liebold and Maas, 2020)

The detection of cracks in probes and the quantitative determination of crack patterns is an important task in material testing. Compared to conventional techniques such as inductive displacement transducers, inclinometers, and strain gauges, which deliver only pointwise measurements, cameras offer the crucial advantage of allowing simultaneous measurements at many locations in an image. Cracks can be detected in monocular image sequences by applying a cascaded image sequence processing chain (Hampel and Maas 2009). Herein, a dense pattern of feature points is tracked by least-squares image matching, yielding subpixel accuracy motion vector fields. These vector fields are analyzed for significant discrepancies, depicting locations of cracks. Metric crack width information can be derived from the discrepancies by a thorough geometric analysis of triangle meshes with the matching points as vertices (Liebold and Maas 2020). The technique can detect cracks with a width in the order of a tenth of a pixel, and it delivers full-field measurements of complete complex crack patterns (see Fig. 3).

The spatial resolution of the technique is mainly defined by the sensor size of the camera, allowing for the detection of complex crack patterns with ca. 100 cracks when using a high-resolution camera. The temporal resolution may reach 100 kHz and beyond. Liebold et al. (2020) applied the technique for the determination of crack propagation velocities in a concrete probe. Image sequences were taken by a high-speed camera at a frame rate of 160,000 images per second. By applying the cascaded image sequence processing procedure to these datasets, crack propagation velocities of about 800 m/s could be determined with a precision in the order of 50 m/s.

A logical future extension of the crack detection and crack characterization techniques shown here is in a transition from 2D image sequences to multi-temporal 3D micro-tomography data. As soon as suitable instruments are available, tomography voxel data acquisition may be integrated into the material testing process.

### Research Perspectives

This section focuses on problems for which the process of solving a problem and finding a solution is not only difficult but also conceptually unclear. This is true for images produced by computational photography, which are usually generated for visual inspection by black-box algorithms. It is currently not known how and even if they can be used for higher accuracy photogrammetric reconstruction, especially if different viewpoints are used. Another challenge is the combination of low-resolution data such as hyperspectral images with high-resolution data in the form of images or 3D point clouds. This is already done for satellite data, but much more high-resolution data from UAVs or the ground lead to additional complications due to the detailed 3D geometry with the associated occlusions.

While traditional flight planning for aerial acquisition is based on regular structures such as grids, a number of challenges come with automating detailed 3D acquisition by means of UAVs in urban areas: as data are more and more acquired in fully autonomous mode, they can be processed in real-time and on-board, opening up possibilities for a more flexible, yet physically feasible, planning of flight paths considering obstacle avoidance, and for online checking of the completeness of the resulting 3D models despite occlusions, incl. instantaneous acquisition of missing image data. A related topic is that of parallel geometric processing, e.g. for bundle adjustment (Mayer 2019).

A particular challenge arises in the overlap between photogrammetry and robotics, i.e., when results are required in real-time, e.g., for docking maneuvers, in autonomous driving, in traffic monitoring, or surveillance. Here, cooperative approaches (Molina et al. 2017; Schön et al. 2018) and swarm processing are considered promising.

Finally, finding robust solutions from image and sensor orientation, as well as dense 3D reconstruction, also as a function of time, remains difficult in scenes with poor or repetitive texture, critical geometric configurations, and/or large depth discontinuities. Examples for challenges in image orientation include the determination of image overlap, e.g., for image sets downloaded from the internet, images taken with a significant temporal difference, or in different parts of the electromagnetic spectrum (e.g., optical and thermal data). Progress has been made for orientation (Kendall et al. 2015) as well as 3D reconstruction (Kendall et al. 2017) by learning matching functions for points (Jin et al. 2021) and shape priors for surfaces. One can expect that the separation between geometry and interpretation (discussed in the next section) will be slowly eroding and at least implicitly more and more information about the specific situation and, therefore, semantics will be included in the up-to-now purely geometric part of photogrammetric image processing. This could be particularly helpful for dynamic scenes and very different viewing directions, whereas for fast-moving objects it might be difficult to track and/or match individual points or patches due to distortions and (self-)occlusions while the objects stay the same. In this respect, also the introduction of motion models to regularize the solution will be helpful.

## Geospatial Information Interpretation

### State of the Art and Case Studies

The core task of automating interpretation lies in the mathematical modeling of topographic or, more generally speaking, scene information, as well as its behavior over time, in combination with its appearance in the data. Topographic information comprises objects such as buildings, roads, and vegetation but also broader categories like land use. Considering other applications, all kinds of objects such as cars, bicycles, pipelines in industrial plants, animals, and persons are of concern. Data range from color to thermal and hyperspectral images to LiDAR and Radar acquired by any of the platforms mentioned before.

While object and scene knowledge necessary for automatic interpretation has been directly encoded by humans for a long time, in recent years there is a strong tendency towards employing methods based on learning by examples. It should be noted that in satellite image classification such machine learning approaches have been used from the beginning; the examples to learn from are training data, which encode the main body of object and scene knowledge, albeit in implicit form. The main advantage of this strategy is that by providing new training samples these methods can be transferred to new datasets or new geographic areas relatively easily.

Concerning methodologies and strategies for learning, there is a strong trend from traditional statistical methods, particularly, graphical models such as Markov Random Fields (MRF) and Conditional Random Fields (CRF), which are now often only used for post-processing, towards all kinds of neural networks. Having been initiated by the large success of convolutional neural networks (CNN) for classifying images just showing one object of relevance (Krizhevsky et al. 2012), they have also been demonstrated to outperform other classifiers in remote sensing applications, partly by a large margin, if a sufficient amount of representative training data is available (Zhu et al. 2017b).

For data with a regular topology such as images, different variants of CNN have been devised, leading to strongly improved results for certain areas. Multiple general ways to improve learning and, particularly, to make better use of the training data have been proposed. For instance, one can focus the acquisition of training data with reinforcement learning, making clear where deficits exist in the results. While this can be efficient, the success depends on the availability of the human operator generating additional intended results, and such approaches are hard to benchmark as the performance can always be improved by another round of training.

#### Case Study “Generation of LoD3 3D Shell Model”

**Fig. 4:**
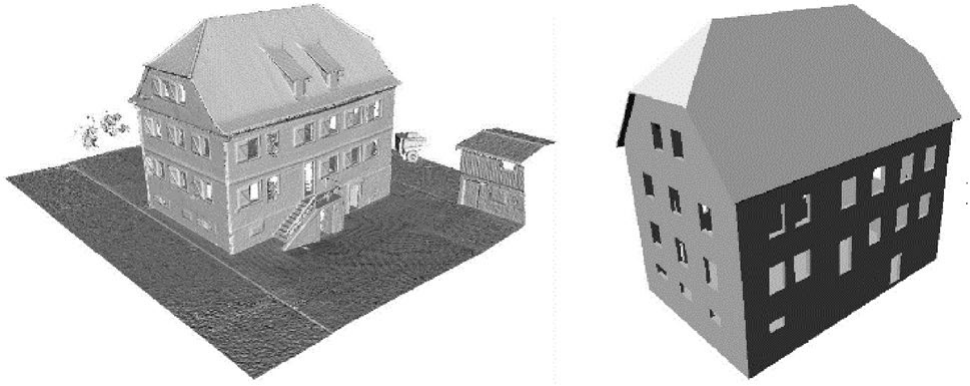
Untextured 3D mesh model (left; see also Fig. 1) and derived LoD3 shell model with roof overhang and holes for windows and doors (right)

Images from the ground and small UAVs, as well as the derived orientations and 3D mesh (see Fig. 1), allow for a consistent reconstruction of whole buildings on the Level of Detail 3 (LoD3): The roof is reconstructed from images from the UAV and the façades including windows and doors from images from the ground. The shell model (Huang et al. 2019) consists of surfaces with a thickness and integrates the roof including overhang and the façades semantically and geometrically (see Fig. 4). This description is derived from the 3D mesh; windows and doors are added as holes. The former is determined from images from the ground projected on the façade planes using a CNN. While the 3D mesh consists of half a gigabyte of data, the shell model just needs a couple of tens of kilobytes.

#### Case study “Scene Flow”

**Fig. 5:**
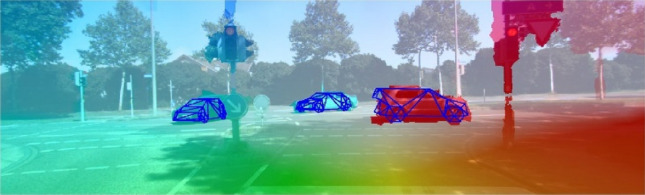
Result for object scene flow (Menze et al. 2018). The reference image is superimposed with optical flow results and the reconstructed objects

Scene flow estimation provides valuable information about the dynamic nature of our 3D environment. In particular, the 3D scene flow field comprises all 3D motion vectors of a densely reconstructed 3D surface model, which is moving with respect to the camera. In their method termed object scene flow, Menze et al. (2018) propose a unified random field model which reasons jointly about 3D scene flow as well as the location, shape, and motion of vehicles in the observed scene. The problem is formulated as the task of decomposing the scene into a small number of rigidly moving objects with attached motion parameters. The inference algorithm then estimates the association of image segments and object hypotheses together with their 3D shape and motion. Figure 5 depicts a sample result of the work.

#### Case Study “Domain Adaptation for Image Classification”

**Fig. 6:**
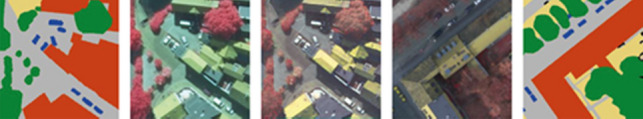
Result of deep domain adaptation using the method of (Wittich and Rottensteiner 2021). From left to right: Classification of source domain image, source domain image, source images transformed to the target domain by the AAN, target domain image, classification of target domain image

Wittich and Rottensteiner (2021) address domain adaptation for the pixel-wise classification of remotely sensed data using CNNs as a strategy to reduce the requirements with respect to the availability of training data. The method is based on adversarial training of an appearance adaptation network (AAN) that transforms images from the source domain such that they look like images from the target domain. A joint training strategy is proposed for the AAN and the classifier, which constrains the AAN to transform the images such that they are correctly classified. A specific regularization term for the discriminator network required for adversarial training helps to prevent the AAN from learning trivial solutions. Using high-resolution digital orthophotos and height data the method on average improved the performance in the target domain by 4.3% in mean F1 score. Sample results of this method are shown in Fig. 6.

#### Case Study “Urban Tree Recognition in Airborne Laser Scanning Point Clouds with Deep 3D Single-Shot Detectors”

**Fig. 7:**
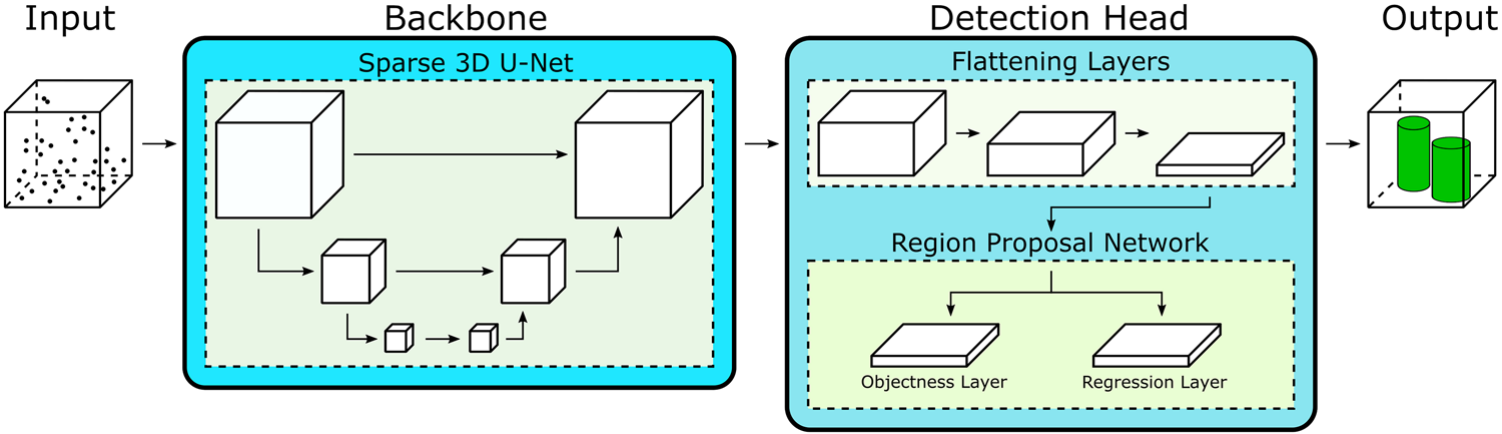
Deep learning network for scene classification and subsequent single tree detection (Schmohl et al. 2021)

To apply CNN for airborne laser scanning (ALS) data processing, often in the first step the irregularly spaced 3D point coordinates and their features are mapped into a voxel grid. However, usually, large parts of the volume are occupied by free space. In addition, due to the sensor principle, ALS data are restricted to capture echoes of those object boundaries which can be reached by the laser signal, whereas the interior volume cannot be accessed. Consequently, such a voxel space is in general only sparsely populated. Therefore, standard convolution schemes are not efficient and lead to a blurring of details, because each 3D point is spread by the filter’s impulse response. Remedy are so-called 3D submanifold sparse convolutional networks that avoid such undesired widening by omitting certain parts of the input (Graham et al. 2018). Schmohl et al. 2021 use such a network as a sparse 3D backbone for feature extraction in a framework tailored to detect single urban trees (see Fig. 7). This vegetation class enjoys steadily rising interest, for example, for purposes like improvement of locale climate or enrichment of biodiversity. Unfortunately, official tree cadasters are often limited to the public ground only, ignoring the large share of urban trees in backyards and private gardens. To detect as many trees as possible, the mentioned 3D backbone is followed by a detection approach, which eventually delivers the height and crown diameter of individual trees.

### Research Perspectives

Geospatial data interpretation has recently profited significantly from artificial intelligence methods in general, and deep learning methods in particular. This development has opened up unprecedented possibilities for new applications, e.g. in combining human interaction and computing performance for active learning, in fusing drastically different images such as terrestrial and aerial views and integrating deterministic physical models with neural networks (Tuia et al. 2021). At the same time, several problems which await sound solutions have become apparent.

A general issue is that the internal decision-making process of deep learning methods is not well understood; they behave very much like black boxes, and there is no theoretically sound strategy for designing deep learning methods for specific applications; instead, mostly adhoc design methods are employed today, starting from some existing architecture that has proven to work well for similar problems. How to explain the behavior of deep learning methods is, thus, an important research question (Roscher et al. 2020).

Another question is how to infer causality from correlation (Pérez-Suay and Camps-Valls 2019). The challenge consists of combining advanced learning methodologies and strategies with a good understanding of the objects, spatial and semantic relations as well as interactions over time, to provide a capable but also ideally provably robust system for interpretation. The problem is aggravated in remote sensing applications due to the large variability of sensors that are used.

A major challenge for the approaches based on learning by example is to provide knowledge via training data, which is suitable for the problem. To improve the generalization capabilities, one has to make sure that the major aspects necessary for interpretation are included in the training data, e.g., the complete spectrum of objects to be interpreted. This all means that there is a new basic challenge: How can one provide training data which contains the relevant variation of the objects and their interactions?

There are large databases of annotated terrestrial RGB images such as ImageNet (Russakovsky et al. 2015) which form a good basis for training classifiers that generalize well for a large variety of applications but are restricted to the object types shown in these images and to the type of sensor used for acquiring them. No datasets of a comparable size exist for remote sensing data, let alone for sensors such as airborne laser scanners or hyperspectral cameras. A new ISPRS benchmark sheds some light on this matter (https://ifpwww.ifp.uni-stuttgart.de/benchmark/hessigheim/default.aspx).

Recent research, therefore, focuses on making better use of self-supervising approaches. A particularly promising way is to employ generative adversarial networks (GANs) for the generation of realistic training data to extend the coverage of the natural variations of objects or scenes. GANs can also be used to learn from non-matching input and output. For instance, mappings between maps and images of the same region can be learned without one-to-one relations.

In particular, in topographic applications, one can make use of existing maps to generate the training labels for new images. In this context, the training technique has to cope with erroneous labels (e.g., due to changes in land cover), so-called “label noise”. Kaiser et al. 2017 have shown that using large amounts of such partly wrong training samples can reduce the requirements for high-quality hand-labeled data in training a CNN. For label noise, robust methods for training can provide good results even without any hand-labeled data, which has been shown for random forest classifiers (Maas et al. 2019). These principles are currently being transferred to the domain of deep learning (Voelsen et al. 2021).

Another very promising strategy is multitask learning (Kendall et al. 2018), where several tasks of possibly different complexity can support each other. In transfer learning and domain adaptation, one learns from previous applications, even when these are only weakly related (Tuia et al. 2016); one of the challenges is to understand how similar the different domains must be for a successful application of domain adaptation, and how this similarity can be measured. In this context, GANs can also be applied to learn a representation of the images so that the resultant features have similar distributions for images from different domains (Wittich and Rottensteiner 2019), or to learn how to adapt the appearance of images from one domain so that they look like images from the other domain (Wittich and Rottensteiner 2021; cf. Sect. 3.1).

The availability of satellite data with high revisit times, e.g. Sentinel-2, has triggered interest in the processing of time series. In this context, recurrent neural networks (RNN) have been used, e.g. for building detection (Maggiori et al. 2017). Long short-term memory (LSTM) architectures were developed to avoid common problems in training, e.g. vanishing gradients (Hochreiter and Schmidhuber 1997), and they have been adopted for the classification of remote sensing data from multiple epochs, e.g. for change detection (Mou et al. 2019) or for crop classification based on time series (Rußwurm and Körner 2018). An alternative is to employ self-attention mechanisms, typically embedded in transformer architectures (Vaswani et al. 2017), which is a more general representation than convolutional network layers (Cordonnier et al. 2020). Such architectures can help to suppress irrelevant observations in time series, as was shown by Rußwurm and Körner (2020): They also reach competitive results in other computer vision tasks (Dosovitskiy et al. 2021; Liu et al. 2022). The achievable geometric accuracy and the amount of supervision required for learning such models, in particular if a pixel-wise classification is to be determined for every time step, still seems to be unclear.

An additional basic question consists in how far geometric and physical modeling is still appropriate. In principle, everything could be learned by just linking the acquired input data to the intended result. While this could be helpful in certain areas with a weak understanding of the problem, such as how to model a tree as seen from the ground with different levels of mutually occluding branches and leaves with a very complex reflection function, experience shows that using proven models as priors tends to improve and stabilize results (see Tuia et al. 2021 and the references therein for examples).

It also has to be noted that learning approaches focus on a semantic interpretation of the input data, but in general do not aim at a 3D reconstruction of the geometric shape of the objects. Some recent examples have tried to support 3D reconstruction by machine learning, e.g., for roof reconstruction (Wichmann et al. 2018) or cars (Coenen and Rottensteiner 2019). It is anticipated that this common treatment of semantics and geometry will increasingly be used and will yield better results.

Similar arguments hold true for dynamic scenes. A general question relates to the usefulness of explicit motion models for objects to be detected and tracked, incl. models for object behavior, which are necessary to predict future situations, e.g. in traffic scenarios, and the interaction between different objects. Also, it is unclear, what the benefits are of modeling not only the behavior of individual objects but the whole scene, in general, as a function of time.

In contrast to raster images, 3D point clouds are irregularly distributed spatial data. To extract implicit neighborhood relations for each point, usually, local features are calculated to enable subsequent segmentation or classification (Weinmann et al. 2015). In addition, for point clouds, substantial progress has been achieved based on deep learning (Griffith and Boehm 2019) employing one of the following processing strategies: (i) use of hand-crafted features in a single- or multi-branch 1D CNN, (ii) projection of the point cloud onto planes which are fed into a standard 2D CNN for images, and (iii) discretization of the point cloud to 3D voxel space, where 3D convolutions take place. A general bottleneck is the lack of sufficient labeled ground truth data for training and validation of such approaches. Furthermore, there is also progress for neuronal networks based on general graph structures (Nassar et al. 2020), also for the direct segmentation of 3D point clouds.

Finally, it will always be necessary to optimize functions. While classical least-squares adjustment or Kalman and Particle Filters allow for a high precision, sampling strategies such as RANdom SAmple Consensus (RANSAC) or Markov Chain Monte Carlo (MCMC) and their many variations are more robust but need to be chosen in a way reflecting the error structure of the problem. The training of CNNs requires the minimization of a loss function. For that purpose, variants of stochastic gradient descent are usually applied. The impact of the chosen optimization procedure on the results has hardly been investigated so far. More work has been spent on designing task-specific loss functions to be optimized, but problems remain, for instance, the way to deal with extremely unbalanced class distributions of the training data, which frequently occur in land cover classification or the development and application of appropriate strategies for regularization to avoid overfitting. Finally, the question arises if and how a combination of robust sampling strategies and CNNs could lead to considerably more robust approaches for automatic interpretation.

## Geospatial information modeling and management

Goodchild (2009) already formulated the goal “imagine the possibility of a world of real-time geographic information”. Today, due to IT and sensor developments during the last decade, we are on the way towards realizing this vision. The challenges presented in the former sections and their consequences to geospatial information modeling and management – driven by expected more data and extremely detailed representations concerning geometry, semantics, extent and time for all kinds of sensors – will lead to new challenges for information modeling and management. In the coming decade, geospatial information modeling and management will have to be adapted to these new requirements.

### State of the Art

A common approach in geographic data science is the integration of multiple data sets characterized by different spatial and temporal references, at multiple scales and resolutions (Andrienko et al. 2017). This means that *information integration* is a central issue bringing together different “dimensions” such as space dimension (2D, 2.5D, full 3D objects: maps and digital terrain models as well as full 3D geological subsurface models and full 3D city models), spatial scale (resolution), and temporal scale (resolution). We will focus on different levels of information integration in Sect. 5. Before integrating data, however, data has to be modeled and managed in a formalized way. A typical example, showing two *different ways of spatial data modeling* which have been established for decades, are the domains of geographic information systems and Computer Aided Design (CAD). In the following, these are generally referred to as geospatial information modeling (GIM) and Building Information Modeling.

Geospatial information modeling denotes the digital modeling method for space-related phenomena of the real world. It is characterized by multidimensional descriptions of geospatial features by location and orientation in a spatial reference system (SRS), object model, and field model (implemented as vector and raster data formats), and has been extended to spatio-temporal models (Erwig et al. 1999; Güting and Schneider 2005). Seen from a historical point of view, in GIM geometric, topologic, and semantic modeling of objects have always been treated as one unit. GIM is used as digital documentation of real-world states and can be applied to a variety of spatially related questions (Herle et al. 2020). Due to the rapid development of information and communication technology as well as advances in the methods and degrees of automation of data acquisition, it has become possible to capture geodata in their 3D or 4D extent and thus to create a very realistic virtual image of the real world in digital form.

Organizations such as the International Organization of Standardization (ISO), with the ISO 191xx family, and the Open Geospatial Consortium (OGC), promote *standards for better interoperability*, including data models (from simple feature model to CityGML, IndoorGML, Land Administration Domain Model (LADM), and Land and Infrastructure Conceptual Model (LandInfra)), exchange formats and languages (such as Geography Markup Language GML) and service specifications (the OGC Web Services such as Web Map Service (WMS), Web Feature Service (WFS), Web Processing Service (WPS), etc.). The resource-centric OGC API family of standards are being developed to make it easy, especially for machines as well as for anyone, to provide geospatial data to the web.

**Fig. 8:**
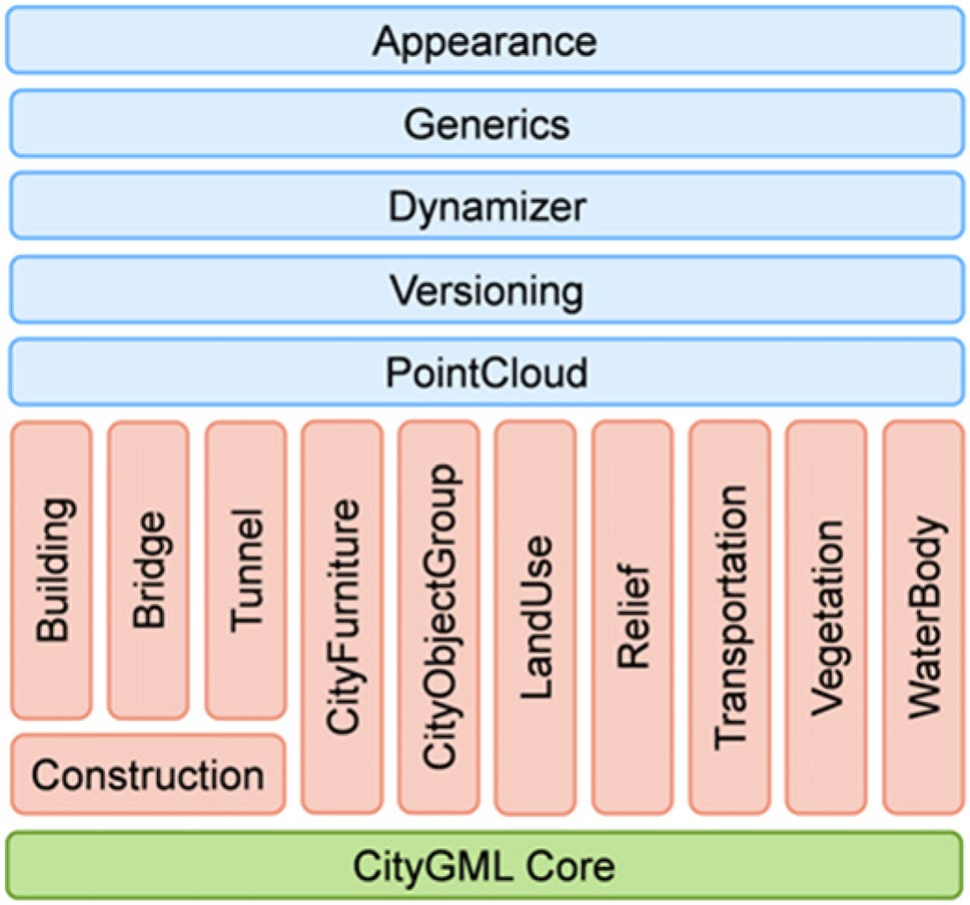
CityGML 3.0 module overview. The vertical boxes show the different thematic modules. Horizontal modules specify concepts that are applicable to all thematic modules (Kutzner et al. 2020)

GIS research, development, and implementation are largely based on CityGML, first adopted as version 1 in 2008 and recently released as version 3.0 (https://docs.ogc.org/is/20-010/20-010.html) in September 2021. CityGML 3.0 is an OGC international standard for modeling, storing, and exchanging *semantic 3D city models*. In CityGML, buildings, terrain, vegetation, street furniture, urban object groups, water bodies, roads, tunnels, bridges, and land use can be represented semantically and geometrically, with the construction module being new (see Fig. 8). In addition to these eleven thematic modules, six modules are defined that are applicable to all thematic modules. Besides the CityGMLCore, Appearance and Generics, which were already available in earlier CityGML versions, the modules Dynamizer, Versioning and PointCloud have been added.

The conceptual schema of CityGML specifies how and into which parts and pieces physical objects of the real world should be decomposed and classified. All objects can be represented with their semantics, 3D geometry, 3D topology, and appearances information. The objects can further be represented using five predefined levels of details (LoD 0–4 with increasing accuracy and structural complexity). The relevant city objects are defined using the Unified Modeling Language (UML); CityGML 3.0 GML Encodings specify an XML schema for the file exchange format. With the construction of large statewide or municipal city models, CityGML has found an important role in the orchestra of OGC standards and is a good base for GI research and development.

**Fig. 9:**
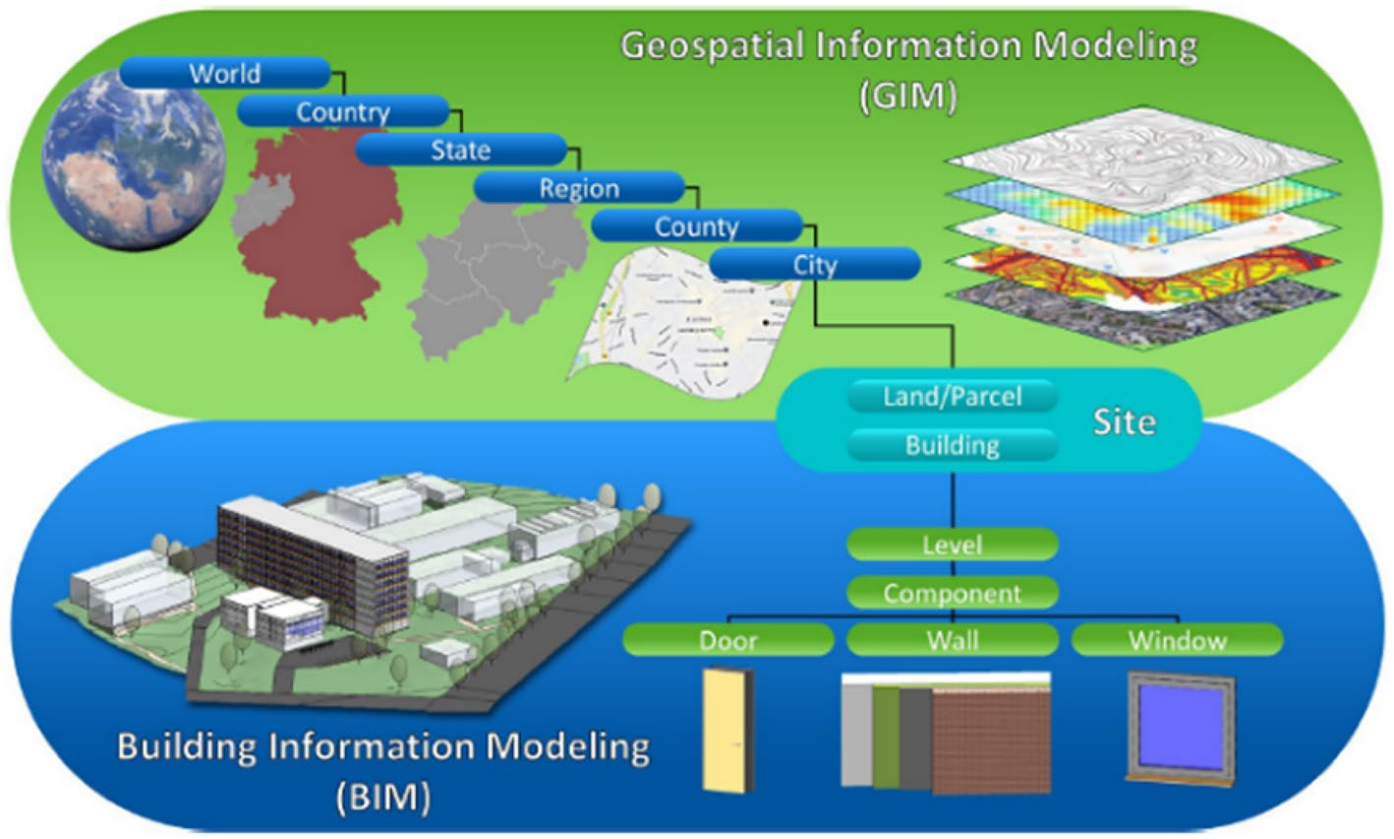
BIM and GIM and their common objects (Herle et al. 2020, adapted from Hutsell and Bush 2016)

BIM and GIM technologies have different origins and come from different domains. In civil engineering and architecture, a shift from purely constructional data handled by computer-aided design software towards *BIM* is currently taking place. This means that the realization of the joint modeling of geometry and semantics has arrived in BIM two decades later than in GIM, still not considering topology as an independent concept besides geometry. BIM (using Industry Foundation Class (IFC) (ISO 16739 2013) by OpenBIM) supports consistent and integrated modeling of all data for specific construction and during its life cycle (Borrmann et al. 2015). BIM and GIM modeling views are complementary to each other. BIM geometry modeling is based on constructed solid geometry (CSG) (Mäntylä 1988), GIM – for instance, CityGML as one of the most relevant standards of OGC for city models—uses boundary representations (B-Rep). Whereas BIM is applied in planning processes for constructing buildings and other structures, GIM is used to model geospatial features of the real world with an application-dependent accuracy (Fig. 9). GIM usually is applied for applications such as heat spread modeling in cities whereas BIM focuses on applications such as facility or energy (heating/cooling) simulations in single buildings which can be up-scaled to urban scale (Geiger et al. 2019). Both concepts are mature and are applied by various industries. Different approaches are investigated to achieve interoperability between GIM and BIM models (Herle et al. 2020).

Information modeling also deals with the development of ontologies at different levels of generality and formality, tailored to various needs and uses (Kokla and Guilbert 2020). Kuhn (2005) defined *geospatial semantics* as “understanding GIS contents, and capturing this understanding in formal theories.” Geospatial semantic modeling and ontologies refer to the meaning of things (Hu 2018).

In the realm of spatial data, linked data and GIS are two separate paradigms, representing different approaches for data representation and exchange. Recently, the volume of data with spatial reference in the *Linked Open Data* (LOD) cloud has been on the rise. Whereas in GIS the main focus is on data for analysis by humans, LOD is structuring information in a format meant for machines. If all datasets were openly published and used the same format for structuring information, it would be possible to interrogate all of these datasets at once. Analyzing huge volumes of data is potentially much more powerful than everyone using their datasets dotted around the web in what is known as information silos. These interoperable datasets are what LOD practitioners are working towards (Iwaniack et al. 2016). Time and space referencing are the simplest methods for structuring such data and providing the context for interpretation. This is one of the reasons for perceiving linked open data as one of the most important approaches for geographic information publication and consumption on the web. It provides new means for sharing, accessing, and integrating geoinformation and holds a promise of changing ways, in which GI developers and analysts solve their problems.

According to Andrienko et al. (2017) “one of the most challenging problems in geographic data science is the need to assess the data quality, suitability, and distribution of the data available for analysis.” The heterogeneity of the real world, different technologies for data acquisition and processing, database management tools and platforms lead to a large amount of duplicated, inconsistent, ambiguous, and incomplete spatial data. Thus, *spatial data quality and uncertainty* is an increasingly important issue in geographic information science. Uncertainty and data quality modeling is an unavoidable part of spatial data due to an approximation of real-world phenomena. The influence of uncertainty may be visible in the form of original data and measurement, assumptions, or in the model structure (Bielecka and Burek 2019).

To allow persistent use of data and objects modeled according to GIM and BIM, geospatial information is maintained in *spatial database management systems* (SDBMS). SDBMS are optimized to store and query data that represent objects defined in a geometric space. They define special data types for geometric objects and allow geometric data (usually of a geographic nature) to be stored either in regular database tables or in non-tabular databases. Besides spatial data types, SDBMS provide special functions and indexes for efficient querying (Güting 1994) and manipulating that data using declarative query languages such as the Structured Query Language (SQL) or others. In the beginning, attribute data were stored separately from the geometry in relational database management systems (RDBMS). With the emergence of object-oriented thinking, complex data types and relations between geometry and topology as well as attributes were treated in object-oriented database management systems (OODBMS) (Balovnev et al. 2004). Object-relational database management systems (ORDBMS) combine both approaches, the relational world, and the object world, and are standard today (Thirunavukkarasu and Wadhwa 2016). These spatial database management systems support at a minimum complex data types, spatial data within related tables—feature classes, feature datasets, validation rules—subtypes and domains, spatial metadata, spatial reference systems and transformations, topologies and methods for analyzing spatial relationships, a spatial query language for query and search, and spatial indexing to improve the performance (Jitkajornwanich et al. 2020).

More types of databases are approaching the market for specific purposes, which are also of interest for geospatial problems: NoSQL databases (not only SQL—more than tables, document-oriented, graph databases) and XML databases (semi-structured data described in XML) are used in many disciplines beyond GIM and BIM (Lee et al. 2012). Furthermore, Content Management Systems (non-structured data, e.g. documents, arbitrary texts, graphics) and multimedia databases (imagery/video/mass data etc.), are well suited to manage the context of geospatial data. Finally, in-memory databases (running in the RAM of the computer, Continuous Query Language), data stream management systems (DSMS) (streams of sensor data, video, audio), and array databases (Baumann 1994) are seeing a revival (Baumann et al. 2021) as there is an increasing need of providing analysis-ready data (Baumann et al. 2018).

### Case Study and Research Perspectives

#### Case Study “Urban Digital Twins”

**Fig. 10:**
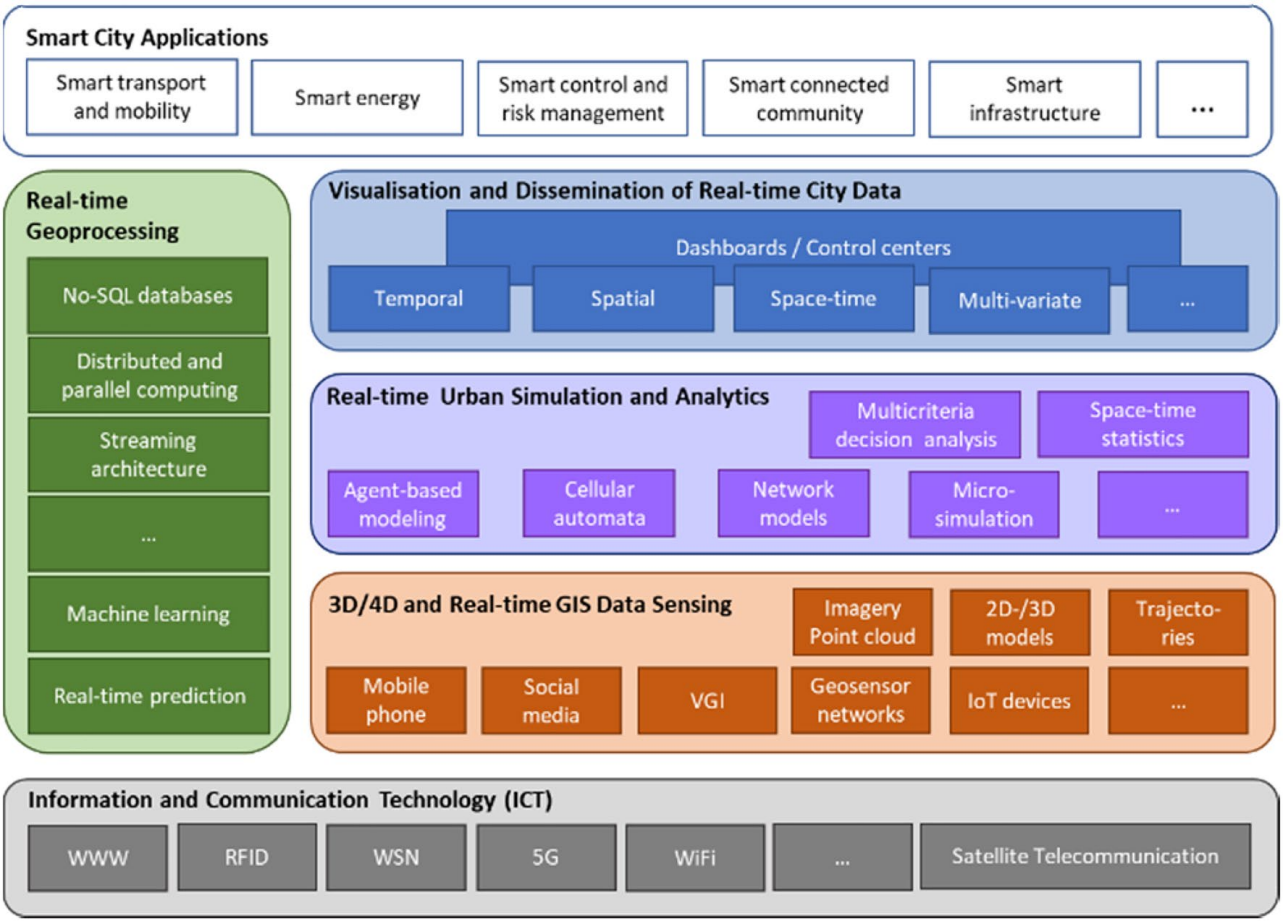
A smart city enabled by real-time GIS (adapted and extended from Li et al. 2020)

Based on the most recent ICT and its protocols a huge variety of 3D/4D and real-time data sources need to be integrated to model our environment. These data, collected by means of both, physical and social sensing, together with modern remote sensing technologies, define what is increasingly called the “digital twin” of a city, available for real-time geoinformation processing. Urban situations should be simulated and analyzed with highly sophisticated mathematical methods for different purposes. Huge amounts of different data need to be stored, maintained and processed in a distributed and parallelized manner. Decision makers, government and the public should be involved, which asks for advanced methods of information visualization and dissemination (see Sects. 7 and 8). It facilitates the integration of urban geodata for a variety of applications for smart cities and urban digital twins, including urban and landscape planning, smart energy, transport and mobility, infrastructure, and others. Li et al. (2020) integrate these research issues in an emerging real-time GIS platform for smart cities that is designed for the acquisition, storage, analysis, and visualization of geospatial data in real time (see Fig. 10). This real-time GIS needs to support high throughput and high-speed processing of large GIS data streams, being location-sensitive, of high temporal granularity, and being generated continuously from sensing devices that collectively comprise the IoT.

#### Research perspectives

Picking up the challenges introduced in the former sections, we now outline the consequences for geospatial information modeling and management in detail. Obviously, new challenges arise such as:

(i) Modeling and management of big geospatial and spatio-temporal data from data-intensive sensors incl. a change from images to videos.
(ii) Representations of buildings, cities, and infrastructures in different space and time scales.
(iii) Modeling and management of full 3D spaces for GeoBIM, i.e. the integration of GIM and BIM.
(iv) Real-time dynamic scenes and moving objects.
(v) Topology in 2D, 3D, 4D, and nD space.
(vi) Ontologies and semantics: Modeling spatial and semantic relations and interactions as well as knowledge about objects and scenes.
(vii) Data quality and uncertainty modeling and maintenance in the whole GI processing chain.
(viii) 4As (anytime, anywhere, for anyone, and anything).
(ix) Database support for big geospatial data analysis.
(x) Artificial intelligence (AI) supporting spatial information modeling and management.

Challenge (i) opens “a new chapter of the book” in geoinformatics: Real-time and highly dynamic scenarios will arise in remote sensing and other geo-applications with a high demand to retrieve and to mine knowledge out of the objects and the scene in (near) real-time. New data stream management systems and array databases, respectively, should be used and adapted to the special requirements of *spatio-temporal data streams*.

The challenges (ii) to (v) indicate that future DBMS will have to be extended to enable the management of multi-scale, full 3D, and topologic objects in *spatio-temporal database management systems* (STDBMS) supporting discrete and continuous time. Full 3D space representations of objects will be also necessary for GeoBIM to combine the data management for GIM and BIM applications e.g. to integrate 3D geological underground models with 3D city models. Besides geometric data types representing point clouds, lines, surfaces, and solids, also topologic data types representing nodes, edges, meshes, and solids should be provided. This means that not only the x, y, z-coordinates of points, lines, and surfaces of solid objects will be represented and ready for export, but also the internal topology of the objects. Furthermore, the semantic properties of these objects can be attached to their geometries and topologies. The topologic representation will make it possible to distinguish between the interior, the boundary, and the exterior of the objects and to navigate through the topologic representations. Furthermore, topologic concepts are well suited to model the relationships between spatial entities such as neighborhoods and intersections. Also, the number of connected components and the number of their holes can be explicitly determined by topologic concepts such as Betty numbers. Concerning time, GIM will require to model not only discrete time (objects only represented in predefined time steps), but also continuous time to model geographic or geoscientific processes such as volcanic eruptions, landslides, or tsunamis, whereas in BIM discrete time steps will be sufficient e.g. to model the progress of a construction site. 4D data representations (3D space plus time) are mandatory to maintain the dynamics of features in the real world. The history and future of objects should be considered equally in future 3D information modeling and management. Doing so, historical data are helpful to model the future by extrapolating the past and simulating the future (Breunig et al. 2020). How important it will be to extend 3D city modeling for archiving the past and planning the future of cities has been portrayed impressively by Matthys et al. (2021).

Big geospatial information, especially the large amount of unstructured text data on the web, and the fast development of natural language processing methods enable new research directions in *geospatial semantics* (challenge vi). Six major areas are identified and discussed by Hu (2017, 2018), including semantic interoperability by developing ontologies, digital gazetteers as structured dictionaries for named places, geographic information retrieval, geospatial semantic web, linked data, place semantics, and cognitive geographic concepts. Elicitation approaches will involve a set of processes that aim at extracting latent knowledge from unstructured or semi-structured content. Future research will be dealing with semantic-based extraction, enrichment, search, and analysis of big geospatial data, places, regions, events, trajectories, and topics as well as geospatial concepts and relations (Kokla and Guilbert 2020).

GI research approaches will integrate *imprecise *geospatial* data models* (challenge vii) such as fuzzy models and rough sets and multiple representations (Virrauntas et al. 2009). Quality models should be able to propagate errors throughout the whole GI processing chain because data imperfections propagated through spatial analysis affect the decision-making process. Thus, error modeling and quality descriptions will be shifted from location uncertainties of geographic features and phenomena, towards fitness for use data evaluation, to uncertainty in decision making (Bielecka and Burek 2019).

Challenge (viii) refers to mobility: “With the integration of information and communication technologies (ICT), especially mobile ICT in every aspect of our daily lives, the *4As (anytime, anywhere, for anyone and anything)* ‘services’ are being developed to benefit our human society and environment. This new generation of 4A technologies brings convenience and improves our quality of life, but also leads to surveillance, privacy, and ethical issues, unknown, and unimagined before” (Huang et al. 2018).

The massive use of geospatial raster data requires efficient database support for big geospatial data analysis (Challenge ix). Therefore, *array databases* (Baumann 1994) such as rasdaman (Baumann et al. 1998) will allow the provision of analysis-ready data as Data Cubes (Baumann et al. 2018; Baumann, 2018) or Earth Cubes (Mahecha et al. 2020).

Artificial intelligence (AI) methods will be included in DSMS, SDBMS, STDBMS, and array databases (challenge x) to support data analysis. Machine learning will likely influence geospatial information modeling and management in a revolutionary way: Future database platforms should be able to deal with the input, models, and output of machine learning approaches to simplify data preprocessing. Furthermore, AI may support data cleansing to detect and correct errors in big data and time series (Breunig et al. 2020). Examples are the tileless spatial and temporal selection of examination areas and the automatic detection of data interpretation errors such as the unwrapping error in Sentinel-1 SAR data (Mazroob Semnani et al. 2020). As a consequence, future scientists will be able to repeat their experiments as often as they like by just retrieving arbitrary spatial and spatio-temporal data from the STDBMS to check and compare the last runs of their experiments or of other researchers who carried out similar data analysis experiments. Combined data analysis and data management tools will thus increase the reproducibility of data analysis results significantly.

Furthermore, the use of parallel data management architectures (MapReduce-based systems) such as SpatialHadoop^®^ (Eldawy and Mokbel 2015), ST-Hadoop^®^ (Alarabi et al. 2018), and Hadoop-GIS^®^ (Aji et al. 2013) and new developments will support fast query processing. New directions in geospatial data management focusing on the interface between different fields of research such as interfacing data management and visualization, data management and data analysis, etc. have been discussed by Breunig et al. (2020).

## Geospatial Information Integration

Geospatial information integration faces a set of challenges related to the high variety of available data in the geospatial domain. While it has similarities with general data integration, data augmentation, and data cleaning, it differs a lot as different conceptualizations and resolutions lead to fundamentally different representations of the same thing in reality. This can lead to situations, where spatial data integration is simpler as opposed to general data integration: When two different objects are in a very similar location, they likely have a strong relationship and the existence of the relationship can be inferred just from geometry and topology. However, fully correct and plausible representations of the same object can be significantly different in terms of geometry: do we identify a house with its perimeter or its cadastral polygon or maybe just with the entrance of the property? Does the garage belong to the house or is it a separate object?

### State of the Art

Data, models, information, and knowledge are scattered across different communities and disciplines, causing severe limitations to current geosciences research (Gil et al. 2019). Geospatial data is captured for different purposes, with different sensors, based on different data models. This leads to a huge variability of available data sets. This variety not only concerns the semantic contents of the data but also to data modeling, formats and representation. As data sets often relate to the same physical reality—or the same spatial and temporal extent of the reality—there is a need for data and information integration to fully exploit the richness of available data. Examples are topographic data sets and special branch data, e.g. ATKIS and GDF; GML and BIM formats, e.g. CityGML and IFC; structured data sets and raw data, e.g. CityGML buildings and 3D building surface models from point clouds.

The goal of data integration is to

- provide a consistent view of a set of datasets from different regimes, schemata, and conceptualizations,
- allow an integrated analysis of data from different sources and
- enrich data sets with information from another one.

Data integration is typically tackled on different levels and with different mechanisms:

- Syntactic integration ensures that comparable data formats are used so that the data can be represented in the same system and e.g. visually overlaid. Such a process can be dealt with using standards (e.g. OGC simple feature specification, CityGML, GeoTIFF).
- Semantic integration relates to the contents of the data and the meaning and aims at identifying corresponding object categories in both data sets. This process presumes that those correspondences are revealed and made explicit. Often, ontologies are used to describe the meaning of objects. An ontology is a shared conceptualization of objects and relations of a specific knowledge domain (Gruber 1995). This means that it is typically a negotiation process among different stakeholders to define and describe what constitutes an object.
- Instance-based integration: Given two datasets with different representations of possibly identical objects, the construction of correspondence of objects in terms of relations like “identity” or “is-part-of” can be used to derive a joint object catalog combining information from both datasets. In geospatial data, such correspondences are typically derived using semantic and geometric matching, i.e. taking similar object characteristics and similar form, shape, and location into account.
- Latent Space Integration: Given a family of independent datasets, learn representations of each of the datasets like a feature transformation (Bengio et al. 2013) and align the resulting feature spaces into a joint space in which the joint geometry captures similarity.

Due to these very different schemes of data integration, the results of the integration are manifold: one option is to derive a consistent, homogeneous object catalog, which integrates and consolidates the properties and attributes of the objects it was derived from. This includes steps such as entity resolution, entity matching, geometric fusion—e.g. deriving an intermediate geometry—and harmonization of the attributes. As all of these steps can be challenging, a fundamentally different approach is to first propagate all the objects from the data sources into a large set, maybe adding the source dataset to the name, or otherwise making possibly identical objects from different datasets to different objects. In this intermediate representation, semantic links between objects are computed via data mining. A good introduction on this topic is given by Getoor and Diehl (2005). These links can be semantic relations such as neighboring, “is-part-of” or “belongs to”. In addition, the “identity” can be mined as a relation “is-same-as”. Furthermore, the certainty of such links can be assigned as a property to the link such that clear relations (e.g., “is-part-of” if the geometric footprints overlap) can be distinguished from weak relations (e.g., “is-part-of” with a weight of 0.5 for two different objects, because the geometry intersects both objects). While this approach avoids errors introduced by conflating two datasets into a new one forcing all information to be merged, it introduces a much more complicated topologic data space as the result of the integration, and it becomes more difficult to implement applications on top of such a data space.

The *semantic web* aims at making the links and relations between data explicit by defining resource description framework (RDF) triples; the field also provides tooling and query languages for the resulting data spaces. However, it is still an area under development and further research is needed to fully unlock the potential of these approaches, especially under the uncertainty inherent to spatial data objects.

In general, data integration in the spatial community is often a process in which at least input and output datasets have a spatial interpretation. An important exception to this rule is the case of latent space methods in which an artificial space (geometric space, topologic space, or differently structured data space) is generated in which a certain problem (e.g., object detection, change detection) can be solved although the original geospatial attributes are (partly) lost in this step. In many such cases, this loss of spatialness as part of the method is compensated by an additional data integration step with the original data. State-of-the-art computer vision algorithms for object detection, like Yolo, are a good illustration of this principle. In these algorithms, the spatial space (e.g., the image) is decomposed into many small patches which are individually processed. For each of those patches, without the location information in the original image, the system assigns a probability that the patch is part of an object to be detected. Then, these partial results are recombined into a spatial object representation like a bounding box. In this case, spatial information is removed in the splitting phase to small patches, and the spatial nature is reintroduced when combining neighboring patches to bounding boxes after classification in a non-spatial image classification setting.

### Case Studies

#### Case Study “Data-driven schema matching”

**Fig. 11:**
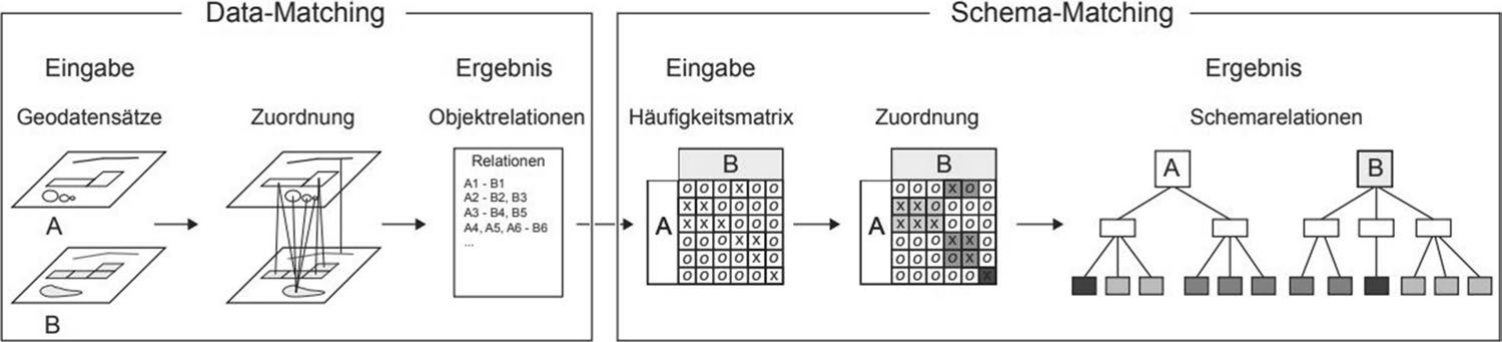
A survey of approaches to automatic schema matching (Kieler 2020)

An approach to achieve semantic integration is proposed by Kieler (2020). It is a data-driven approach to determine correspondences between two ontologies and starts from the assumption that objects which share the same geometry and spatial footprint also have something in common on a semantic level (see Fig. 11). An example is a forest, which is called „A4107 (Wald, Forst)“ in ATKIS and “G7120 (Land Cover: Forest (Woodland))” in GDF, and which in both data sets is represented at the same location and with a similar geometric delineation. If there are many examples of such correspondences, then it is fair to infer that “A4107” and “G7120” belong to the same semantic class. Based on this principle, Kieler developed an optimization approach using integer programming to determine the best correspondences between two data sets. The challenges are that typically the correspondences between objects are not unique (i.e. not 1:1 but 1:n or n:m) and there are geometric inaccuracies. In addition, when objects are represented in different scales, the identification of correspondences has to be refined and has to go beyond geometric matching, taking the changes into account that result from generalization.

#### Case Study “Data Enrichment Based on Information Fusion”

Crowdsourcing based on ubiquitous sensors has a huge potential for collecting dynamic environmental information. An approach exploiting smartphone sensors mounted on bicycles to determine road roughness is described by Wage and Sester (2021). The approach is based on the intuitive principle, that roughness can be measured using the acceleration sensors of a smartphone. In this way, the roughness of a road segment is observed by different bicycles.

**Fig. 12:**
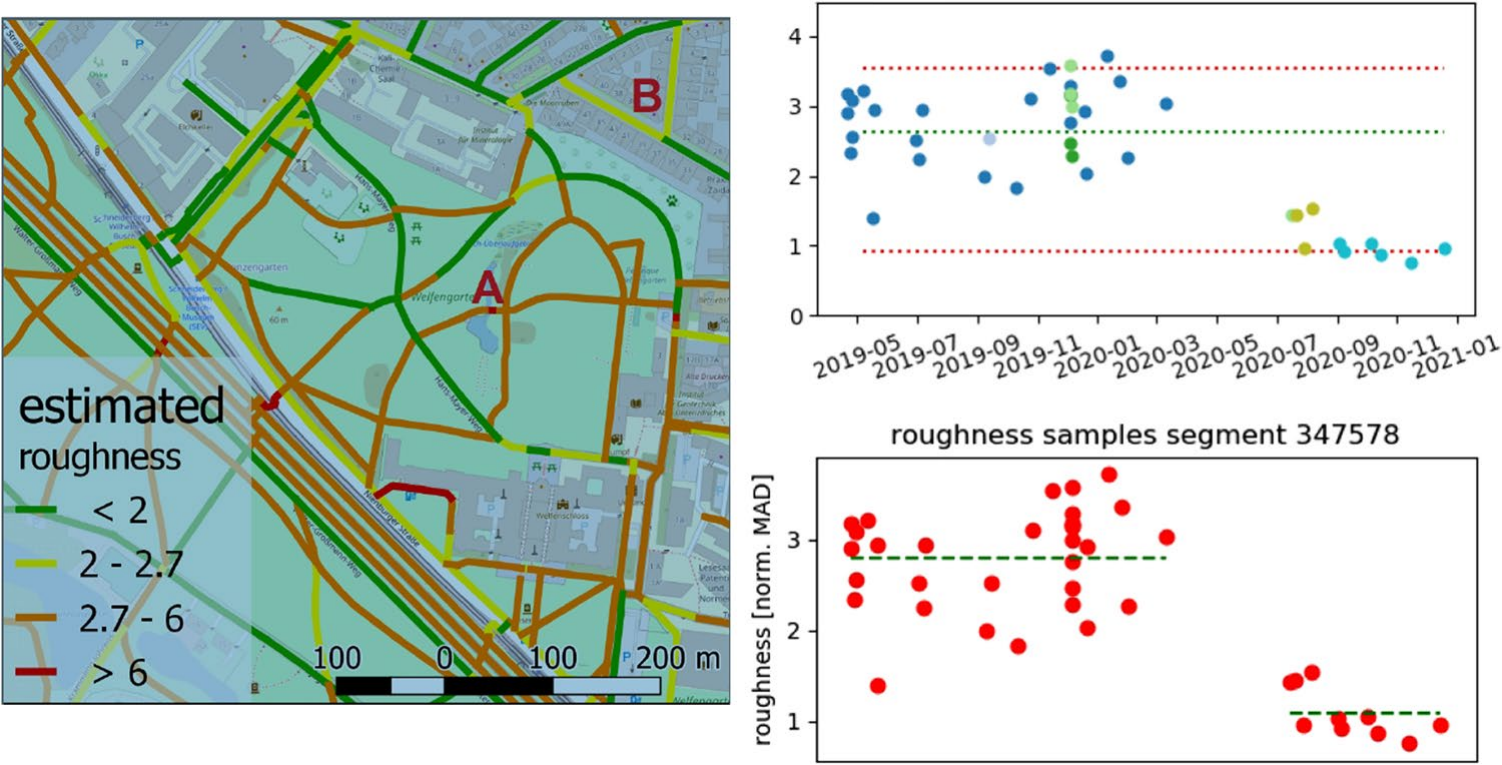
Result of joint estimation of roughness (left); detection of temporal changes in roughness (right) (Wage and Sester (2021))

However, due to different influences such as the type of bike and setup, the observations will not yield the same roughness values. To incorporate parameters describing the context of measurements, the authors set up an adjustment model and jointly optimize the unknown roughness and the unknown context variables (describing for example the damping of the bike) exploiting the measurements and knowledge from the map. In this way, all the observations of the same road segment contribute to the unknown roughness value of a segment, under the constraint that all observations must yield the same value. The problem is solved by a least-squares adjustment, so in parallel to the segment roughness also parameters representing the main varying influencing factors are estimated. In the first step, the trajectories of the cyclists are matched to a road network; then for each road segment, the trajectories are treated as an observation. After the adjustment, roughness values for all road segments are estimated (see Fig. 12 left). The approach assumes that the roughness is constant for each segment—which, however, is not necessarily true over time, due to degradation or renewal. Thus, it is possible to analyze the adjusted roughness observations over time and automatically check for temporal discontinuities (see Fig. 12 right).

#### Case Study “Integration of Spatial Feature Maps Extracted from Social Media with Remote Sensing Images for Urban Heat analysis”

**Fig. 13:**
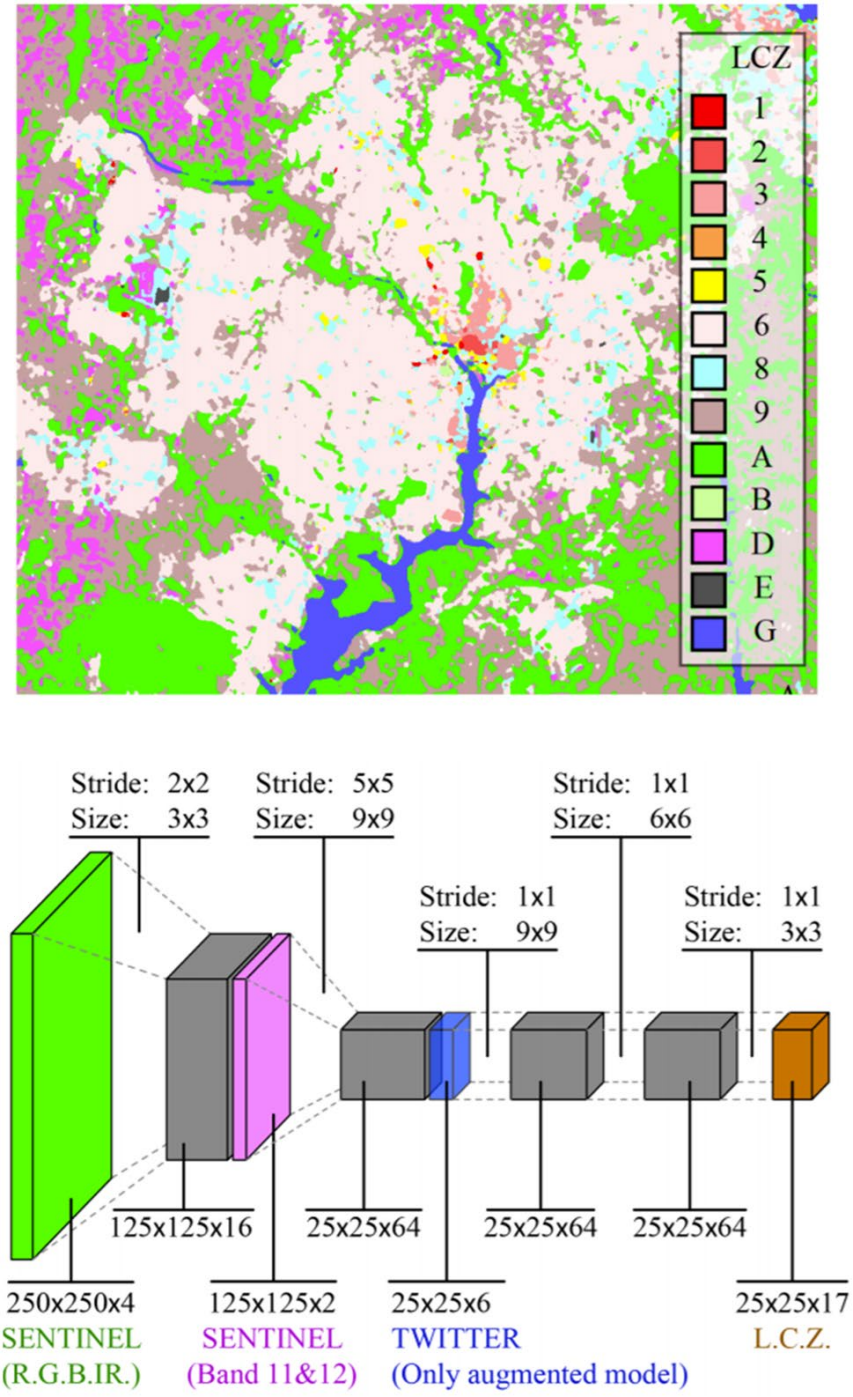
Local Climate Zone Data Example and Deep Data Integration Architecture: Different data sources are integrated into their “natural” resolution by adding the information in the correct “location” of a typical CNN (Leichter et al. 2018)

This is an example of the combination of spatial information of various types into a single deep learning model learning a joint representation of the input datasets suitable for classifying urban regions in a certain resolution (Leichter et al. 2018). The underlying model of local climate zones (LCZ) describes urban settlements in several classes including aspects such as the density (compact, open, sparse) and the height (low-rise, mid-rise, high-rise) of the buildings as well as some additional classes for the non-built area (dense trees, scattered trees, soil, water). Combinations of these features lead to a classification scheme with 17 classes (Steward and Oke 2012). This classification scheme, however, also defines a scale and proposes to assess these classes on a scale comparable to a 100 m × 100 m grid. In this example, the authors combine social media data from the Twitter social networks (e.g., georeferenced objects with metadata from the user account and spatial information from the spatial distribution of tweets) with satellite imagery from the Sentinel-2 mission, which includes various spectral bands and different resolutions. In terms of information integration, the authors start with a spatial input dataset represented by Sentinel-2 layers for red, green blue, and near-infrared (R.G.B.IR.). These are all given in 10 m resolution in a certain UTM projection. Then, the authors applied elements of typical CNNs like pooling and inject all given information in their natural resolution. For the misalignment in terms of projection, nearest-neighbor interpolation is used for Twitter data and bicubic interpolation for images. Figure 13 depicts the architecture: After considering the first layers (R.G.B.IR.) at 10 m resolution, pooling is done to generate 20 m resolution and band 11 and band 12 (which have this resolution) are injected by stacking them onto the feature maps already extracted. A further pooling brings us to 100 m x 100 m in which the authors sample the average number of friends and comparable features from all Twitter messages observed in this area for a three-month interval. Then, some convolutional steps allow the model to transform until the output shall be the LCZ map for the given patch. As the initial patch size, the authors use 250 × 250 pixels, that is patches of 2.5 km × 2.5 km (see also Fig. 13).

This is an example of data fusion in which a single deep learning model (feature learning) is used and data of different nature (features extracted from Twitter, different resolution image bands, etc.) are combined in a joint model learning to predict the local climate zones. In total, this approach increased the performance as measured by the F1-score (a measure of a test's accuracy ranging between 0 and 1) by 0.05 across all classes with significant margins of up to 20% for some of the classes.

#### Case Study “Model Fusion as a General Data Integration Technique”

The previous example has shown that deep learning and other machine learning techniques based on learning representations are helpful because these representations can be combined (by stacking information into feature maps, for example) in the context of a problem to generate spatial output combining various non-integrated data sources. As a final case study, Hoffmann et al. (2019) show the case of ground-aerial fusion. Given a machine learning problem (e.g., building function classification in residential vs. commercial or similar), aerial imagery of the area of interest and street view information, how can one predict the function of a building (or detect damages, or generate map information)? In this situation, even the feature spaces that one can generate during deep learning do not align well with each other as aerial images show information that is significantly different from street-level information (e.g., roof shape vs. façade), but still informative for the result (e.g., building function). In this situation, the authors showed that combining features in a single end-to-end learning paradigm does not work well. Instead, the authors solve the problem for each input dataset separately and treat the output not as deterministic information, but as probabilistic votes. That is, the authors train a probabilistic classifier independently on each of our non-integrated input datasets trying to solve the problem directly. The resulting probabilities can then be combined, in the simplest case by taking the mean, and the joint classifier outperforms classifiers based on a single optimization in most cases.

**Fig. 14:**
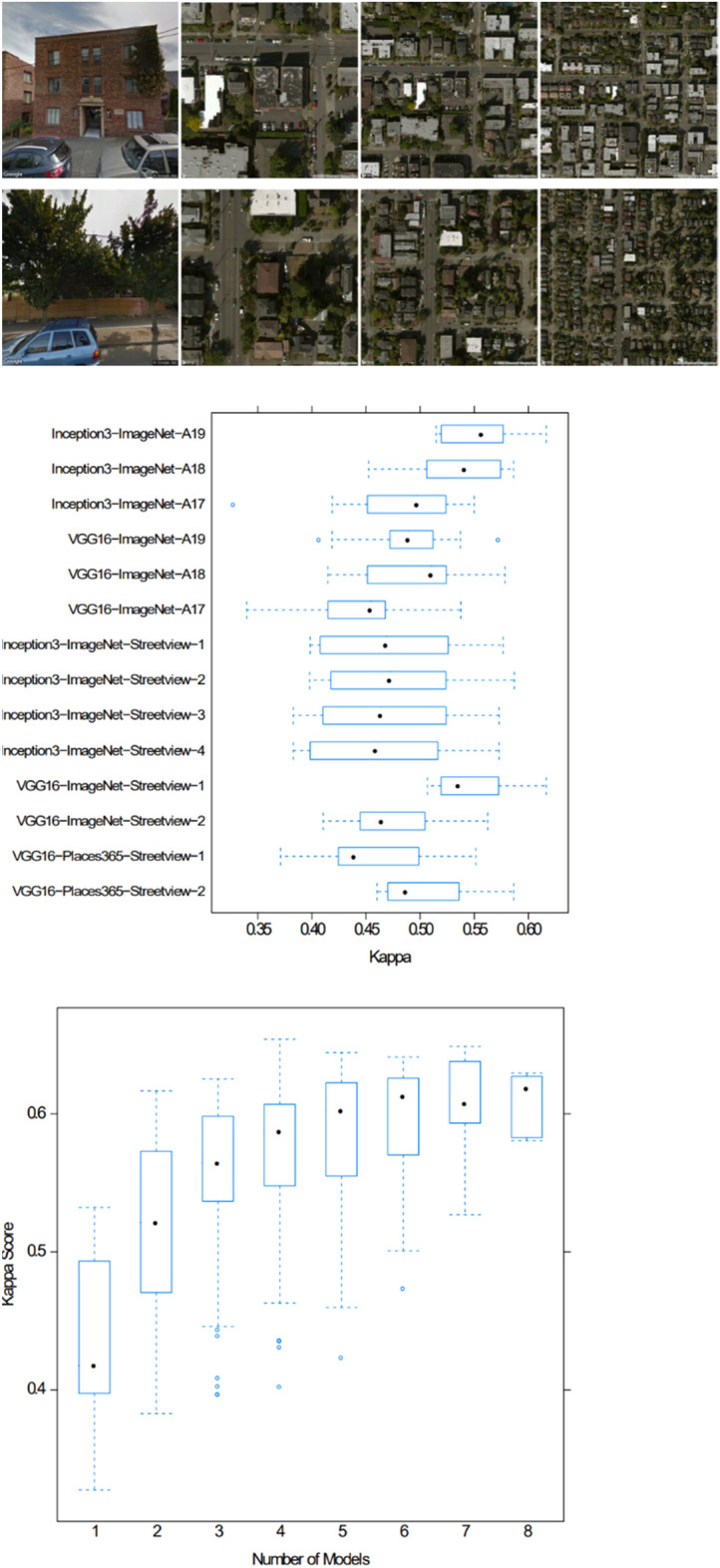
Decision-level fusion for multimodal data integration: As Streetview and aerial images do not share any geometric features, they are treated independently and combined systematically by decision-level fusion (Hoffmann et al. 2019)

Figure 14 (top) depicts the non-integrated dataset on the left-hand side: three resolutions of overhead information and a Streetview image of the same location are combined. In Fig. 14 (middle), one sees the performance of individual models combined with other models through a mean of the output function.

That is, the distribution depicted in each row of this figure is the distribution of performance of combining the named model with any other model in our experiments. The model name consists of the architecture (Inception, VGG), the pre-training dataset (ImageNet or Places365 for Streetview models), and a counter as the authors trained different models for each combination. One sees that all these combinations are not extremely powerful, that high-resolution (A19) overhead imagery is the strongest feature, but that Streetview can help as well. Finally, Fig. 14 (bottom) shows the reachable performances of taking the mean of up to eight models. Increasing the number of (different) models, the average performance increases and the variance decreases. This leads to a better overall classification and another example of that diverse teams can be stronger than well-trained teams lacking diversity.

However, this strategy relies on the fact that each model contributes independent information, that is, when applying it to data that is more correlated than Streetview and overhead imagery or scaling the number of models to even higher numbers will not necessarily lead to better results.

### Research Perspectives

The ultimate goal is to automatically integrate information of any two data geospatial sets into a joint framework, which allows for integrated data analysis. This implies that correspondences on an instance and schema level are automatically detected, deviations between the data sets (e.g. differences in geometry and/or in semantics, e.g. higher granularity, level of detail) are identified. This enables future systems to automatically select the correct data source (or combination of data sources) for any problem at hand.

According to Gil et al. (2019), open issues include cross-domain concept mapping, entity resolution, scientifically valid data linking, and effective tools for finding, integrating, and reusing data. In the following, a few of these research challenges are briefly addressed.

#### Analysis Ready Data

This concept assumes that all relevant information about individual data is available in terms of metadata descriptions. An object “knows” about its properties and also for which processes it can be used. Such knowledge can, e.g., be modeled in terms of knowledge graphs (Auer and Mann 2019). In this way, it is also possible to leave data in their raw form until they are needed in a different representation. An example is 3D point clouds, which are an adequate representation for many applications, e.g. for visualization, determination of clearance in buildings or roads. For other applications, however, a detailed and rich semantic and geometric model might be required, e.g. for planning an extension of a building. Under the paradigm of analysis-ready data, such models could be derived on-demand and on the fly.

#### Information Integration and Information Quality

In general, when applying data integration over spatial data sources, one consequence is that the data quality will change. Unfortunately, it is very difficult in practice to quantify such information losses or gains in advance and independent of a concrete application. This is why data integration is usually performed for each application individually from a set of base datasets which are rather pure observations. By providing information products together with their “lineage”, that is, how they have been created and how parameters have been chosen and tradeoffs have been resolved, information products could be designed that have a more universal applicability in data fusion.

#### Scale and Generalization

When information of different spatial scales is integrated, the aggregation effect of generalization has to be considered: through generalization, objects are combined, simplified, and they might change their topologic relations with other objects. Thus, typically the data has to be transformed to a comparable generalization level before they can be integrated (and possibly generalized further jointly) (Hampe et al. 2004).

Furthermore, problems of scale and also round-off errors can have effects such that even the coordinate transformation between two different datasets or the integration of two datasets of different spatial scales can have an influence on the outcome of very simple operations such as point-in-polygon filtering. Keeping track of these errors in all processing stages and especially keeping track of the error propagation is crucial for reliable complex workflows based on data integration. However, tool support for such quality-oriented data integration is still largely lacking and it relies mainly on the experience and care of the data scientists performing the data integration.

#### Linking Data and Processes

Traditional geoinformatics often concentrate on data and its automatic analysis. On the way to more and more automation, however, methods for linking the data with applications and tasks are needed. This can be achieved by introducing more knowledge on different levels: on the one hand knowledge about the data, their semantics, and relations; on another level, however, also knowledge about processes and connections between different knowledge chunks and underlying physics or rules. Knowledge graphs are one way to formalize this knowledge (Mai et al. 2020). Furthermore, it would be good if data was always accompanied by its whole processing history, metadata, and associated software tools. Only in this way, spatial analysis can be reproducible and causes of problems can be identified.

#### Machine Learning and Data Fusion

In the last decades, machine learning has become a more and more mature technique. One way of using machine learning for data fusion and data integration is to use machine learning to project data into a data space in which the distance or topology has some application-dependent meaning. For example, one can learn a vector representation of text in which sentences with similar meanings are near each other. A good introduction to this topic including high-quality open source implementations is given by Facebook’s fasttext library (Joulin et al. 2017). Such models have lately been applied to various spatial data types as well. The basic idea is that we want to construct a so-called latent space in which a notion of similarities like the Euclidean or the cosine similarity of feature vectors captures a, maybe noisy or only partially available, notion of similarity or identity in the source datasets.

#### Heterogeneous Information Networks

Spatial data integration inevitably leads to situations in which ambiguities need to be resolved and, as discussed before, we can either resolve them by data imputation, data selection, data integration, or random choice. Alternatively, we can make these ambiguities explicit in a linked data representation based on knowledge graphs. However, spatial data models are often formalized in a relational or object-oriented schema (e.g., objects are of certain types and these types have certain required or optional attributes). Knowledge graphs, in their general form, neither represent nor constrain data to follow such schemata and, hence, if they are used for data integration, such schema information is lost. Heterogeneous information networks have been proposed as an approach to generalize the notion of a database schema to knowledge graphs and to render more flexible the queries at hand in such a way that schema information can be exploited. In this case, every node in the knowledge graph is assigned a certain type, and types can prescribe subgraph patterns such as which relations should (at least) exist and how they are modeled. This schema information (type and attribute information) can be valuable in efficient querying and data completion or imputation tasks. Furthermore, such schemata can be enforced implying that all modeled data in the knowledge graph contain all required information for an application. However, these techniques are still not fully mature, and spatial aspects have not been at the center of investigation yet. And the challenge of finding and integrating different schemata is not solved by the concept. Heterogeneous information networks can, however, be a viable tool in data integration as the existence of schemata on the input and the output can be exploited (Shi et al. 2016).

Many more research challenges need to be resolved to reduce the problems that spatial data integration currently still implies for practitioners. These challenges relate to aspects like user-friendliness, explainability, real-time properties, cost, complexity, communication, and many more. In summary, data integration methods can be roughly categorized as follows:

(i) Methods that *resolve ambiguities and enforce a clean data output* at the risk of errors, loss of information, and loss of quality.
(ii) Methods that *avoid to enforce a clean output* at the expense of an increased complexity and the risk a need for query languages, and joint conceptualizations.
(iii) Methods that have a *non-spatial but simple intermediate representation* (e.g., vector embedding, deep learning data fusion) at the risk of loss of explainability, generality, and of limited usefulness beyond the initial use case.

Future research should reduce the number of errors occurring in methods of type (i), reduce the burden on practically working with complex linked data in methods of category (ii), and provide more universal representation learning schemes in data fusion research to make (iii) a more useful data integration strategy beyond representations tailored to single applications.

Drawing a final conclusion, we can say that despite the fact that geospatial information integration provides a well-studied and long-formulated problem, no one-fits-all solution has been proposed so far. Due to the complexity of the tradeoffs involved, geospatial data integration will remain to provide a vast number of research opportunities including *case studies* (examples, how one can successfully integrate into a certain limited setting), *foundational computational research* (novel algorithms and data models to mitigate certain challenges and incompatibilities existing today in the geospatial field), and *accompanying multidisciplinary research* (societal, political, economic, and ethical considerations in the context of data integration).

## Geospatial decision support

### State of the Art

A *spatial decision support system* (SDSS) can help spatial planners and decision-makers conduct an analysis of the situation and come to appropriate decisions. SDSS is defined as “interactive computer-based systems designed to support a user or group of users in achieving a higher effectiveness of decision making while solving a semi-structured spatial decision making problem” (Malczewski 1999). In this context, a “semi-structured” problem is a problem whose criteria are not well defined a priori, e.g., due to a lack of knowledge or because of different stakeholders having different objectives. SDSS combines spatial and non-spatial data, the analysis and visualization functions of GIS, and decision models in specific domains (Crossland 2008; Keenan and Jankowski 2018). According to Sugumaran and DeGroote (2011), an SDSS usually consists of five components, including a geographic information system, a model management component, a dialog management component, a knowledge management component (including a knowledge base and an inference engine), and a stakeholder component (including methods and tools supporting the involvement of and communication among different players).

A crucial and challenging step towards tackling a spatial decision-making problem is, thus, to develop a mathematical model that formalizes the problem’s criteria in terms of constraints and potentially competing objectives. Another step is to compute an optimal trade-off between these objectives subject to the constraints of the problem. Often, the aim is to compute a set of alternative solutions to the problem (rather than a single one) to reflect different preferences among the objectives. Moreover, spatial decision-making is usually considered as an iterative process, e.g., since an evaluation of the solution may reveal the necessity to revise the model.

The types of decision problems addressed with SDSSs include resource location-allocation (e.g., a decision concerning which land to allocate to realize a building project), network routing and reachability (e.g. the best path from A to B or service coverage areas), resource status decisions (e.g., a decision concerning when to harvest a field), and policy decisions (e.g., a decision concerning subsidies to promote wind energy). A more detailed introduction is provided by Keenan and Jankowski (2018).

### Case Studies

#### Case Study “Land Use Options and Ecosystem Services”

**Fig. 15:**
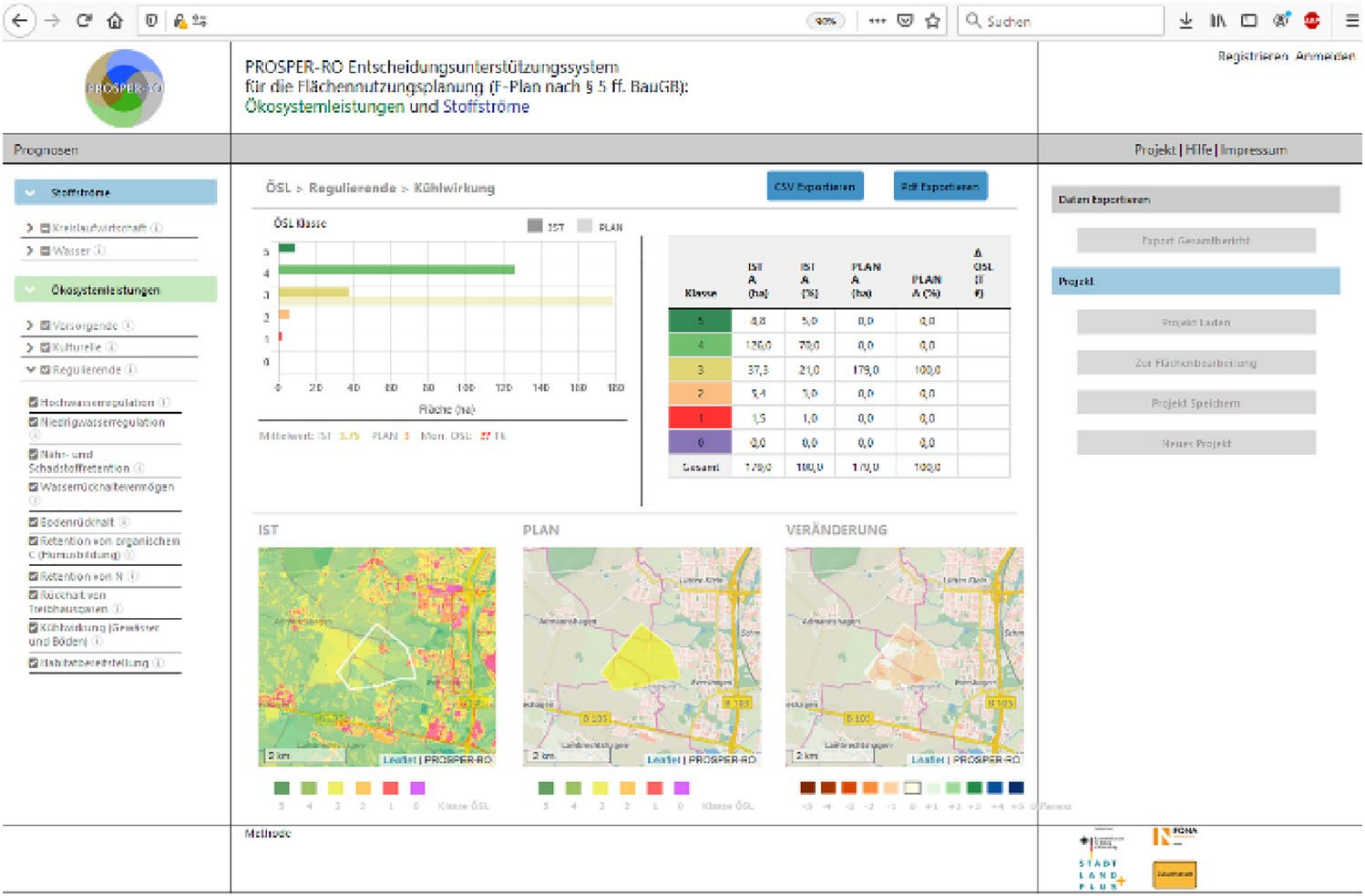
GIS-based decision support system for land use options and ecosystem services (Source: Project PROSPER-RO)

Land is a limited resource. It is under increasing pressure from competing uses such as urbanization, agriculture, forestry, and mining. Therefore, decisions involving the use of land have to be well informed and balanced. The aim of PROSPER-RO (Prospektive synergistische Planung von Entwicklungsoptionen in Regiopolen am Beispiel des Stadt-Umland-Raums Rostock, https://prosper-ro.auf.uni-rostock.de/) is to strengthen cross-regional and cross-actor cooperation in the Rostock urban-surrounding area through i) providing a GIS-based expert support system (GIS-EUS) as a jointly used data and planning basis, ii) developing a uniform monetary valuation standard for all land functions based on the ecosystem service approach and iii) using the aforementioned products to develop concrete synergistic solutions for the areas of land development, circular economy and water management. The decision support system (see Fig. 15) developed in collaboration between science, administration, and private enterprises uses a rule-base. The implementation is based on open source software and integrates authoritative and open data (Hoffmann et al. 2021).

#### Case Study “Public Health Monitoring”

**Fig. 16:**
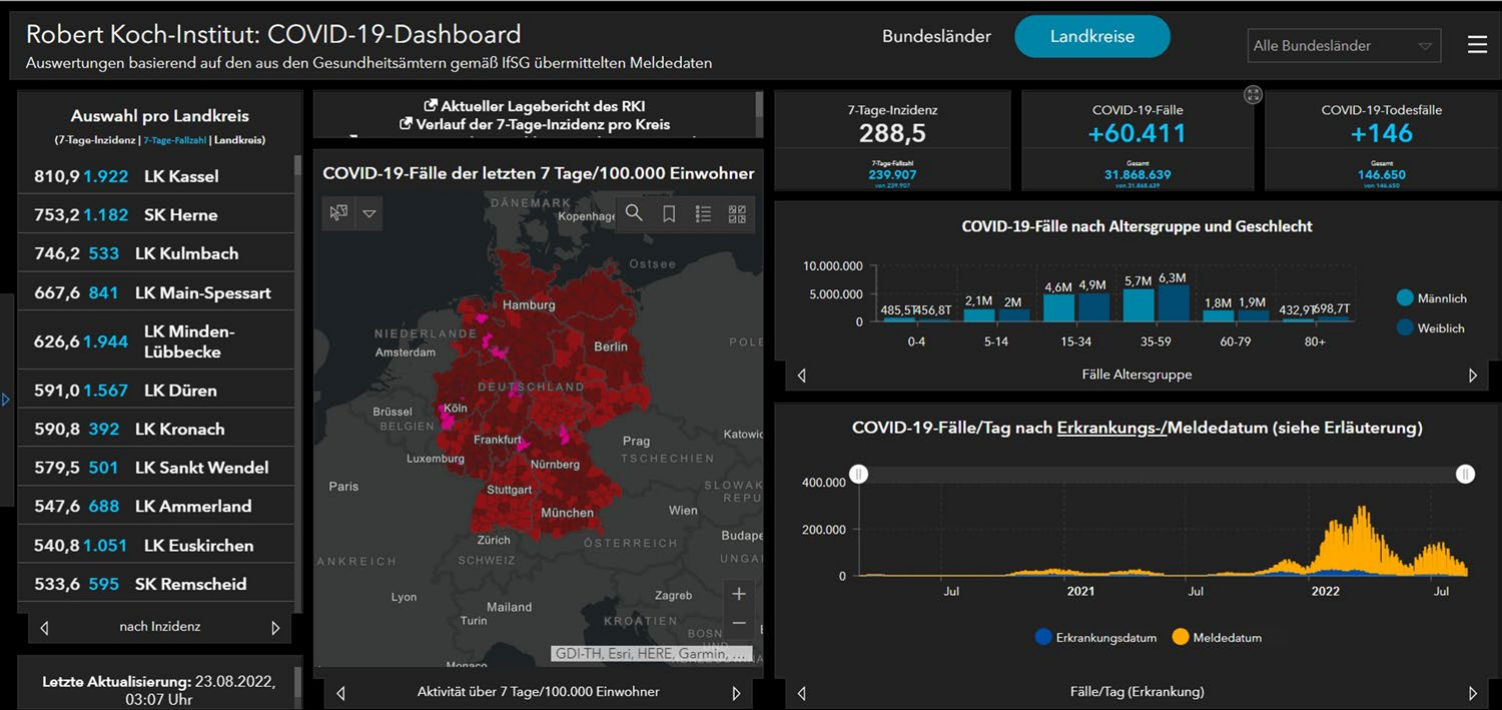
COVID-19-Dashboard of the Robert Koch Institute (https://experience.arcgis.com/experience/478220a4c454480e823b17327b2bf1d4)

The implementation of SDSS has the potential to facilitate public health decision-makers with many tasks from detecting high-risk locations for influenza outbreaks to determining the distribution of medical facilities (Beard et al. 2018). The importance and potential of such decision support systems for disease surveillance by incorporating spatial and temporal components of reportable disease data to model outbreaks and using geographic information systems for analysis and visualization is currently encountered by everybody every day in the Corona pandemic (Fig. 16).

#### Case Study “Decision Support for Smart Farming”

**Fig. 17:**
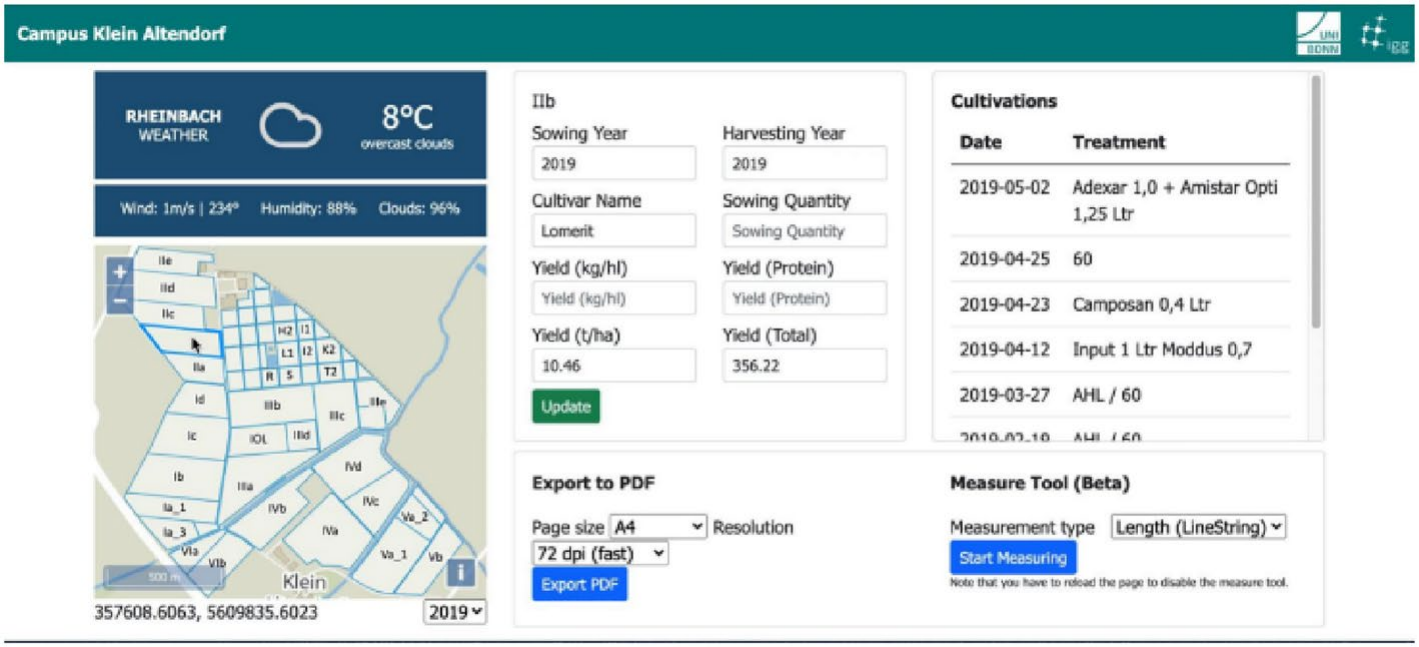
A mobile app developed in the cluster of excellence PhenoRob that shows an interactive map, weather information, and information from the PhenoRob database, such as information on past crop treatments (Source: Project: PhenoRob). (Image taken from a mobile application, leading to a reduced resolution)

Robots and unmanned aerial vehicles equipped with multiple sensors are increasingly used by farmers and agricultural scientists for the monitoring of field crops. Based on the data acquired, decisions concerning field operations (e.g., weeding or fertilizing) are inferred. The aim is to increase the precision of such operations and, consequently, the sustainability of crop production. While future field robots and UAVs will reach a high degree of autonomy, human experts will still be indispensable, e.g., to plan the mission goals of a robot. This requires a good awareness of the current situation. Therefore, mobile apps that provide an overview are needed. Figure 17 shows a solution that has been developed in the cluster of excellence PhenoRob (see Sect. 9) to help human experts make the right decisions.

### Research Perspectives

The application fields of SDSS will increase, e.g. in the environmental management of waste and pollution, in dealing with natural hazards, emergency, and disaster responses, or in forestry and agriculture applications, just to name a few. Due to the close link between SDSSs and GIS, the development of the two goes hand in hand. However, certain challenges of geodata acquisition, representation and data fusion, analysis, and visualization are of particular importance in the context of SDSS and need further research.

With the advent of the *IoT and big data*, the problem of semantic heterogeneity caused by different meanings of data, terminologies, and models needs to be solved.

Methods to analyze urban and landscape structures are needed to provide spatial planners and decision-makers with a better understanding of the situation. The emerging field of *urban analytics* focuses on analyzing urban data (e.g., geographic and demographic data) to detect patterns that are relevant for city planning tasks. A typical example is the analysis of data on urban green spaces together with census data and data on transportation networks to detect areas with a shortage of green spaces (Oehrlein et al. 2019).

*Simulation methods* are needed to predict the growth of a city (e.g. cellular automata), changes in land use, and to determine event patterns by agent-based modeling. For this task, modern machine learning approaches offer new opportunities. Furthermore, tools supporting *multi-criteria decision-making* including new *multi-objective optimization* algorithms embedding the fundamental (i.e. special) properties of spatial data, spatial dependence, and spatial heterogeneity are needed (Malczewski and Rinner 2015; Malczewski and Jankowski 2020). This relates, for example, to algorithms for spatial unit allocation, land use allocation, or districting that, today, often produce only one single optimal solution as an outcome. Developing algorithms that yield a set of alternative solutions is a challenge for future research.

The timely availability of spatial data requires both, advanced planning in the provision of appropriate SDI data and the use of crowdsourcing and real-time data collection to augment or update existing data. Integrating the huge amount of *sensor data or social media data* becoming available worldwide will allow focusing SDSS research on *real-time decision-making in mobile or dynamic environments*. Mobile devices will facilitate the delivery of results of SDSS for location-based services such as decision support for the public or specialized applications in, for instance, smart farming. Moreover, as highly detailed *3D city models,* as well as real-time data from IoT sensors and social media, are becoming increasingly available, spatial planners have, on the one hand, new opportunities to make more informed and timelier decisions but, on the other hand, also need new methods that can handle large sets or streams of heterogeneous and sometimes fuzzy data.

New *visualization solutions* are needed to provide decision-makers and the public with a good overview of the current situation of their environment. Dashboard-style visualization systems (as shown in Fig. 16), for example, are a promising tool for real-time traffic monitoring. Furthermore, interactive web-based multi-user geoinformation systems are needed to enable discourse among different stakeholders and to support public participation.

New methods and models should enable decision-makers in business and public policy to respect given constraints in strategic planning and to understand geospatial contributions and impacts (location matters!). Research is needed to determine the right spatial units for analysis and defining the boundaries at an appropriate local scale, but consistent with a common global basis. This requires the development of an accepted system of metrics that can be applied consistently at and across *different scales*. New modeling approaches should allow the integration of *fuzzy knowledge* by knowledge engineering techniques. New decision-making techniques will involve research on *spatial thinking, analytical reasoning, and knowledge engineering*.

Heterogeneous and conflicting multicriteria decision analysis (MCDA) can be applied to consider multiple stakeholders’ points of view, as well as the multiple aspects of the problem under examination. This allows the stakeholders to express their preferences to decision criteria and/or alternative scenarios using GIS-based procedures, which provide feedback, increasing the trust in the results. *Public participation SDSS* represent a demanding and advanced application of SDSS principles supporting the public having a real interest in decisions made affecting their local region. This brings together people from different social and educational backgrounds, and this diversity poses a challenge to represent the decision problem in a format suitable for the public which may have had no training in spatial representations. Web-based applications may be appropriate, including interactive elements such as *geo-questionnaires and geo-discussion platforms*, in which questions and structured online discussions are coupled with interactive analytics and visualizations to collect data on people's preferences concerning current and/or future conditions. Moreover, those maps become *‘visual indices’* offering solutions to the planners to change and optimize the conditions and support collaborative decision-making involving spatial representations and data sets.

In the future, decision support will benefit even more from *mainstream IT developments and societal demands*. The development of suitable cloud-based tools will provide the computing capacity to address problems with large amounts of data. Traditional government and commercial sources of spatial data have been augmented with open data initiatives (starting from OpenStreetMap in 2004 via open governmental data to crowdsourced data today) or geolocated social media data. Standardization and service orientation support easier integration in classical administrative and enterprise environments (Keenan and Jankowski 2018). The general IT development will allow the integration of computationally intensive models and to run more complex scenarios. Richer sources of data and increased complexity only increase the decision-support challenge of presenting information in a way that reflects the needs of decision-makers. This will foster research on rich problem representations, including the use of dashboards with multiple GIS layers linked with graphs, tables, text and/or 3D visualization.

## Geospatial Information Visualization

The task of visualizing geographic information with maps is classically attributed to *cartography*. At present, however, it is often addressed with a highly interdisciplinary approach.

### The Interdisciplinarity of Geoinformation Visualization

Today, it is common to consider problems of cartographic visualization in the context of information visualization, which also includes the visualization of non-spatial information with other means than maps, such as diagrams or charts (Andrienko and Andrienko 2006). This modern view of cartography has led to the term “geovisualization”. Information visualization provides the link to computer science and several of its subdisciplines, including computer graphics, human–computer interaction, and theoretical computer science (with a focus on the development of efficient geometric algorithms and data structures), as well as to cognitive science and psychology, which are particularly relevant for the evaluation of visualization by users. The development of interactive visualization systems for the analysis of large volumes of spatio-temporal data is often subsumed under the term “visual analytics”. Moreover, present-day cartographic research often involves experts from application domains, such as geoscience or social sciences. Last but not least, experts in geodesy and geospatial information science deal with cartography while considering the whole process of map production from the acquisition of data to the dissemination of maps. In particular, they bring in expertise in modeling geographic phenomena with spatio-temporal and thematic data models as well as generalizing and integrating the respective data while considering the uncertainties in them.

Often, cartographic research is driven by technological innovations that entail both new opportunities and challenges. As an example, smart and mobile devices have enabled a whole range of new applications of maps for location-based services. Cartographers have understood this, on the one hand, as an opportunity to generate new types of personalized and interactive maps addressing a user’s individual need in his or her current situation. On the other hand, providing a good overview of the situation on the small screen of a smartphone or smartwatch is still a largely open challenge.

### Case Studies

This section aims to shed light on current and future challenges of geovisualization by discussing some recent research works of exemplary nature.

#### Case Study “Visualization of POIs”

With the increasing availability of point data—for example as user-generated POIs or measurements with huge sensor networks—the problems of enormous volume and semantic and temporal heterogeneity must be addressed. Both aspects can drastically reduce the user-friendliness in the visual representation and exploration, in particular through geometric or thematic point cluttering. There is still a significant need to optimize generalization workflows designed for specific high-level visual interpretation tasks. Static, multi-scale, or multi-temporal points must be taken into account. One possible solution considers the task-oriented generalization of these point data sets using agent-based modeling (Schiewe 2019).

#### Case Study “Intuitive Visualization Based on Mixed Reality”

Due to more and more hardware solutions and falling costs, Mixed Reality (MR) is finding its way into everyday life. Although 4D geodata is an essential component in virtual or augmented reality applications, cartography and maps have only played a subordinate role in the entire development process so far. For example, generalized maps that are linked to other visualizations can be used for the overview function in MR. Collaboration and communication between MR users need to be improved, which includes further development of interfaces as well as on-the-fly and collaborative generation of map elements such as symbols or annotations (Edler et al. 2019). Additionally, gamification elements have already shown a positive motivation effect.

#### Case Study “Visualization of Uncertainties”

Diverse uncertainties due to errors in raw data acquisition, uncertain model assumptions, or misleading visual representations are rarely taken into account in spatial analysis workflows. To be more effective and efficient, the visual representation should not reflect the detailed representation of individual values, but the effects on decision-making. This requires a more detailed, task-oriented concept of visualization formats and parameters that are tailored to specific tasks. This also includes the often neglected aspect of uncertainty propagation (Knura and Schiewe 2020). Closer cooperation with cognitive sciences is necessary because the cognitive processing of uncertain information still is a fundamental and not well-understood problem.

#### Case Study “Visualization-Based Spatial Cognition”

**Fig. 18:**
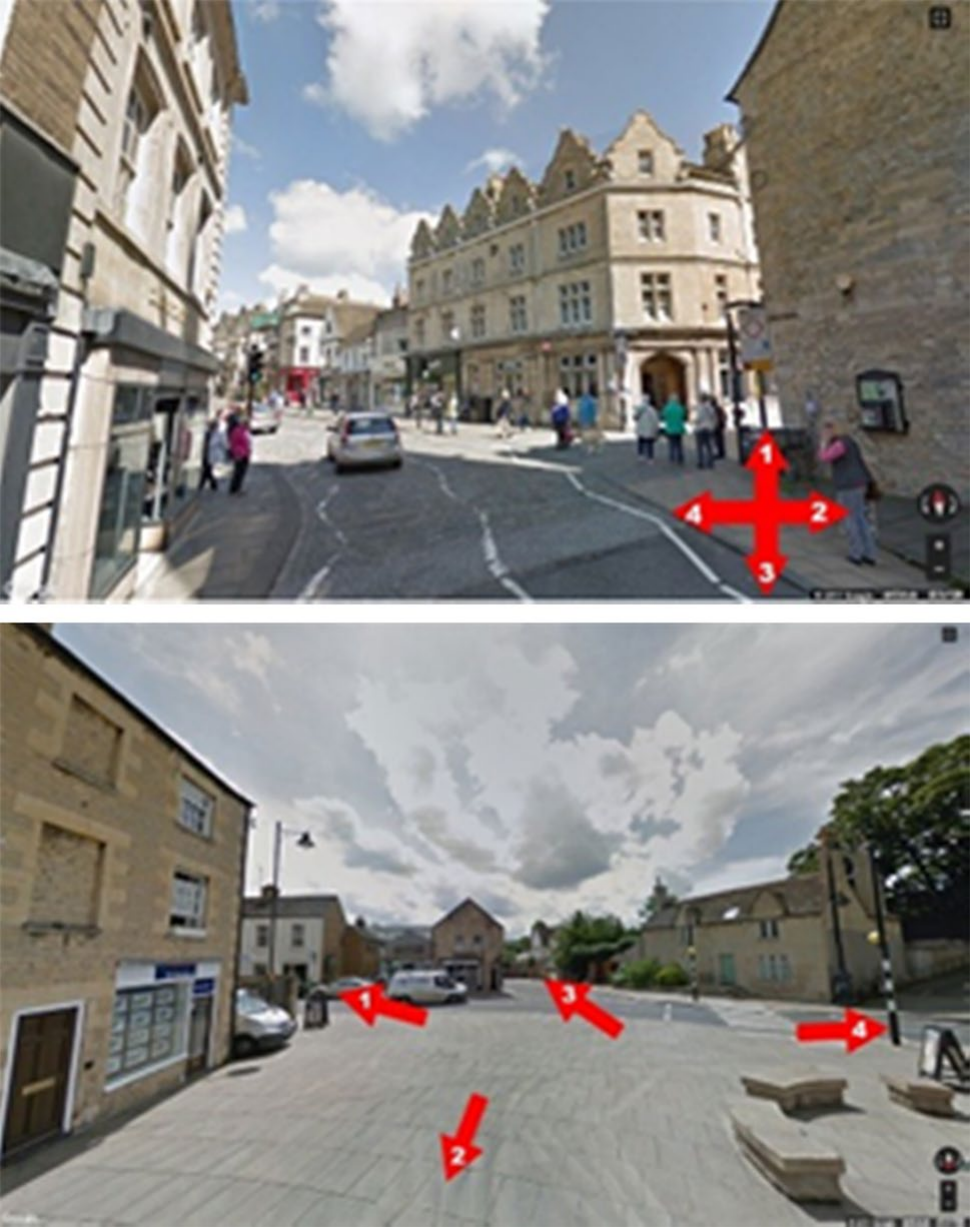
Test setting for orientation (top) and shortest route selection (bottom) (Liu et al. 2020)

**Fig. 19:**
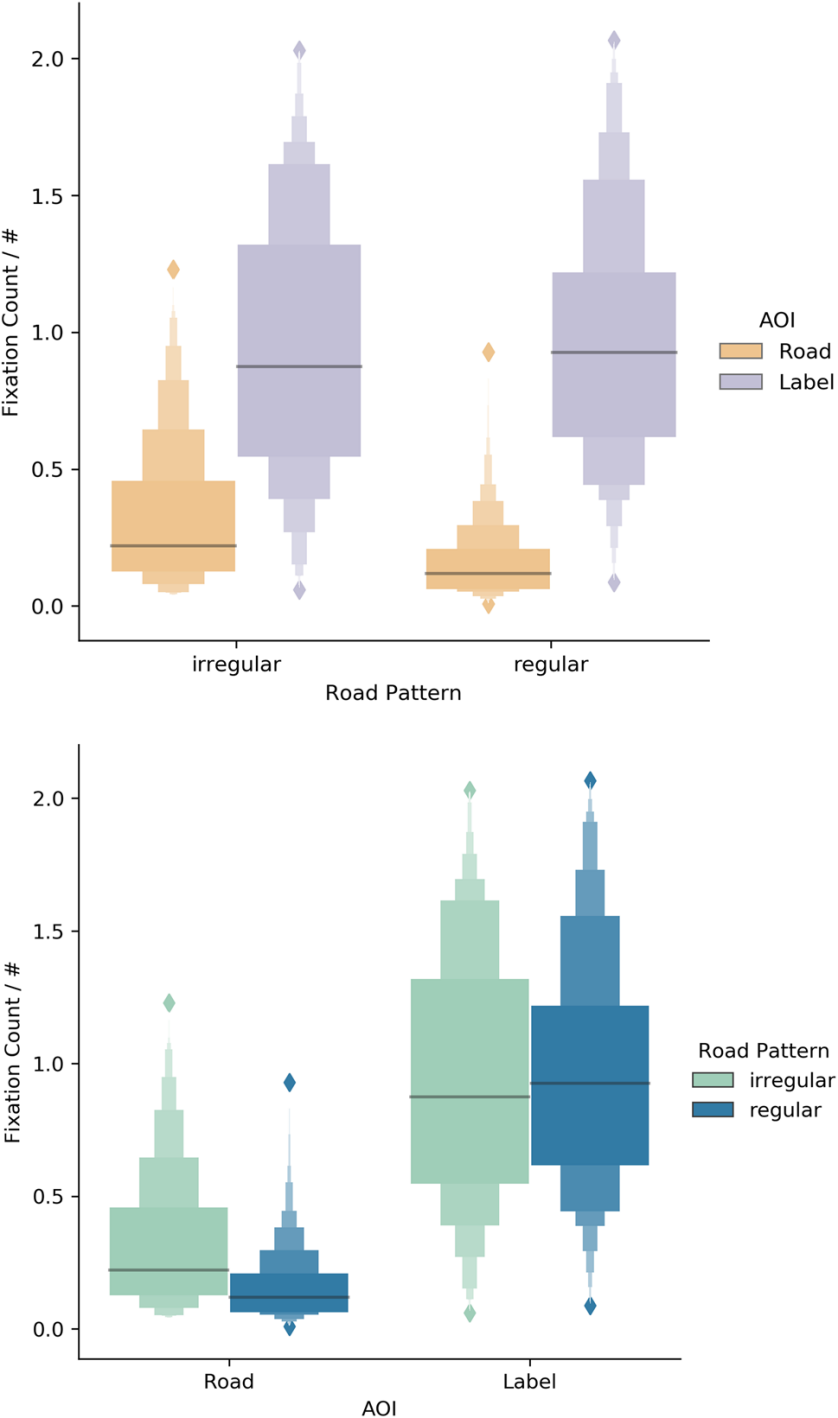
Fixations of orientation for two road patterns (left) and two landmark categories (right) (Liu et al. 2020)

The eye-tracking technologies enable the documentation of eye movements for spatial cognition tasks. Two case studies are reported here. The first one has the objective to explore the impacts of road patterns and landmarks on navigation and wayfinding based on the gaze behavior of pedestrians. Given regular and irregular road patterns in Streetview images, landmarks on roads and outside roads, 21 users were recruited to perform tasks of orientation and shortest route selection (Figs. 18 and 19) (Liu et al. 2020).

The analytical results showed that participants using irregular road patterns relied on landmarks more strongly and performed better in terms of more accurate answers than those using regular road patterns. Moreover, all participants regardless of tasks and road patterns rely on labels or landmarks in the environment more intensively than those on the roads.

**Fig. 20:**
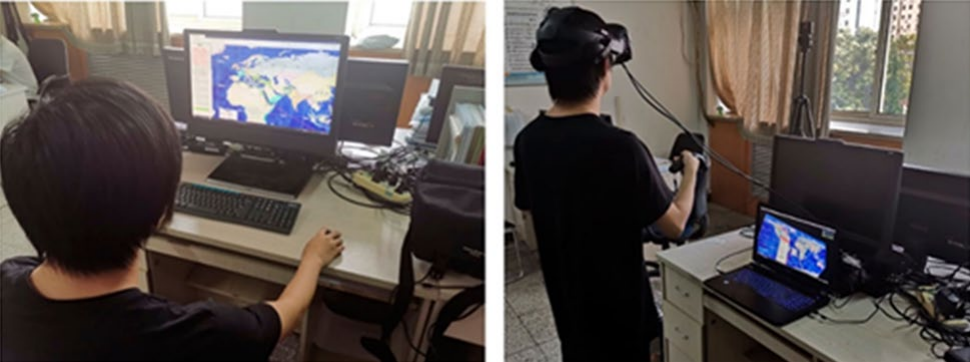
Desktop Environment (left) versus Virtual Reality (right) (Dong et al. 2020)

The second case is dedicated to exploring the performance of map use in a desktop environment (DE) in comparison to virtual reality (VR) as shown in Fig. 20 (Dong et al. 2020). A total of 108 participants were recruited for three groups of tasks: estimation, ranking, and association. The performance indicators include accuracy, information search, information processing, interaction, and subjective rating. The analytical results of eye-movement patterns have proven the complementarity between DE and VR.

There are no significant differences in terms of accuracy and satisfaction; participants of VR processed information faster than their counterparts in a DE, but the other way around for the search tasks. The ranking task requires the most interactions, but the estimation is most difficult in both environments. In addition, the performance of map use is more heterogenous among participants of VR than those in DE. This implies the need for habituation for VR. These user tests can be transferred to larger scales involving outdoor test environments and neuroimaging technologies such as electroencephalography and functional magnetic resonance imaging.

#### Case Study “Interactive Maps for the Exploration of Spatio-temporal Point Data”

**Fig. 21:**
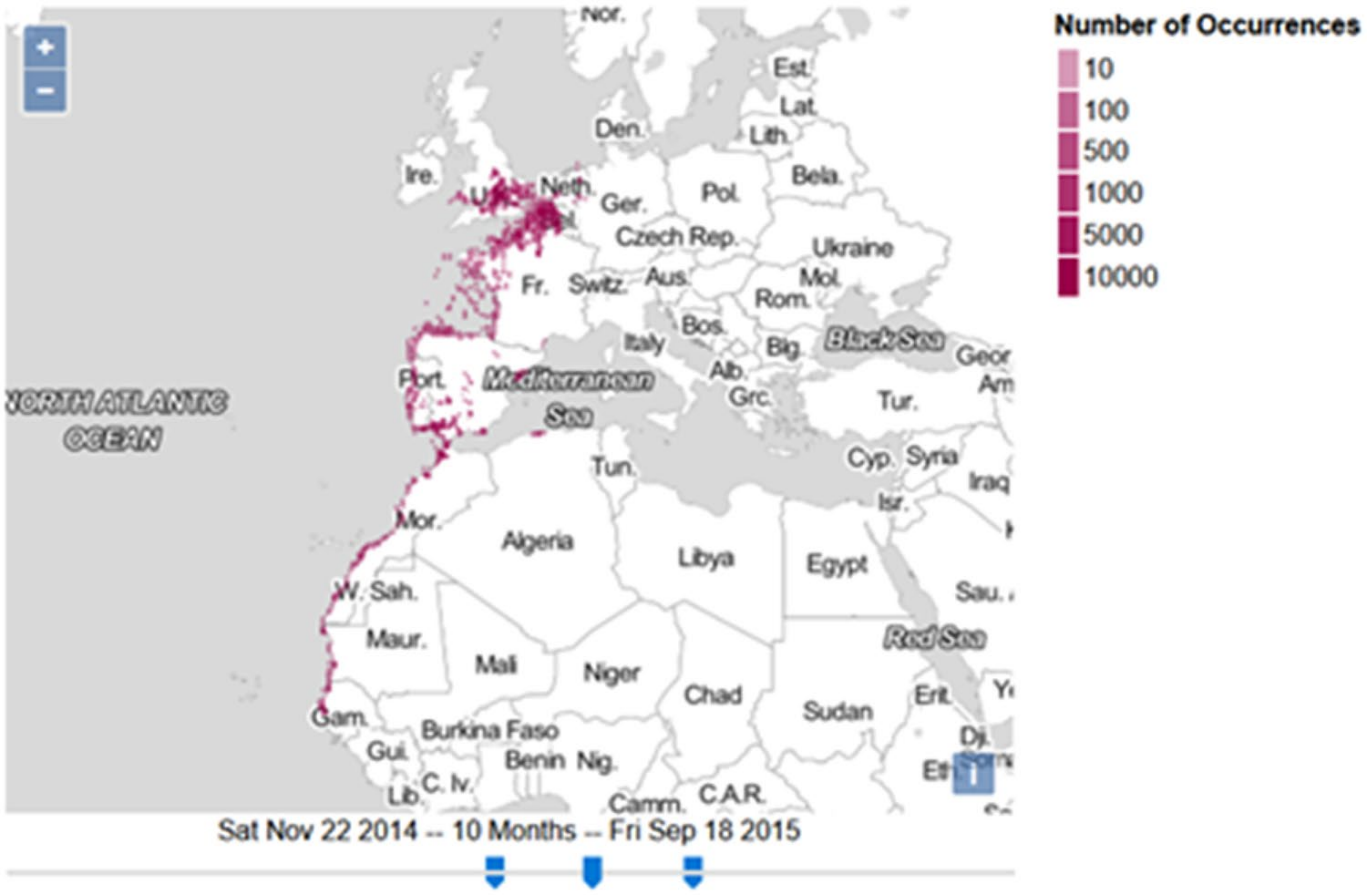
Number of reported occurrences of gulls within a selected time window (Image taken from a mobile application, leading to a reduced resolution)

Contributions uploaded to social media or crowdsourcing platforms often include both spatial coordinates and time stamps. As a consequence, large collections of geo-tagged events have become available. Examples are data sets about occurrences of birds reported by voluntary bird watchers as well as visits to attractions reported by tourists. Effective interaction techniques are needed to enable the discovery of spatio-temporal patterns in such data sets. A common interaction technique is to filter the data with a temporal window and to render a map providing a good overview of all events within the filter. Efficient methods are needed to speed up the map generation in this scenario, such that a user can continuously move a temporal filter over the time axis and, at any time, sees the map corresponding to the current position of the filter without a noticeable delay. To solve this challenge, an idea is to adopt the concept of time-windowed data structures that was introduced by Bannister et al. (2014) in the context of fundamental problems of computational geometry. Bonerath et al. (2019) developed an efficient time-windowed data structure for maps showing polygonal representations of clusters of events within a user-selected temporal filter. Similarly, Bonerath et al. (2020) presented a time-windowed data structure to efficiently generate a spatial density map for all events within a temporal filter (see Fig. 21). This research shows the potential of specialized geometric data structures for the interactive exploration of big geodata and the need for closer collaborations between cartography and computational geometry.

#### Case Study “Cartographic Label Placement for Small-Screen Maps”

**Fig. 22:**
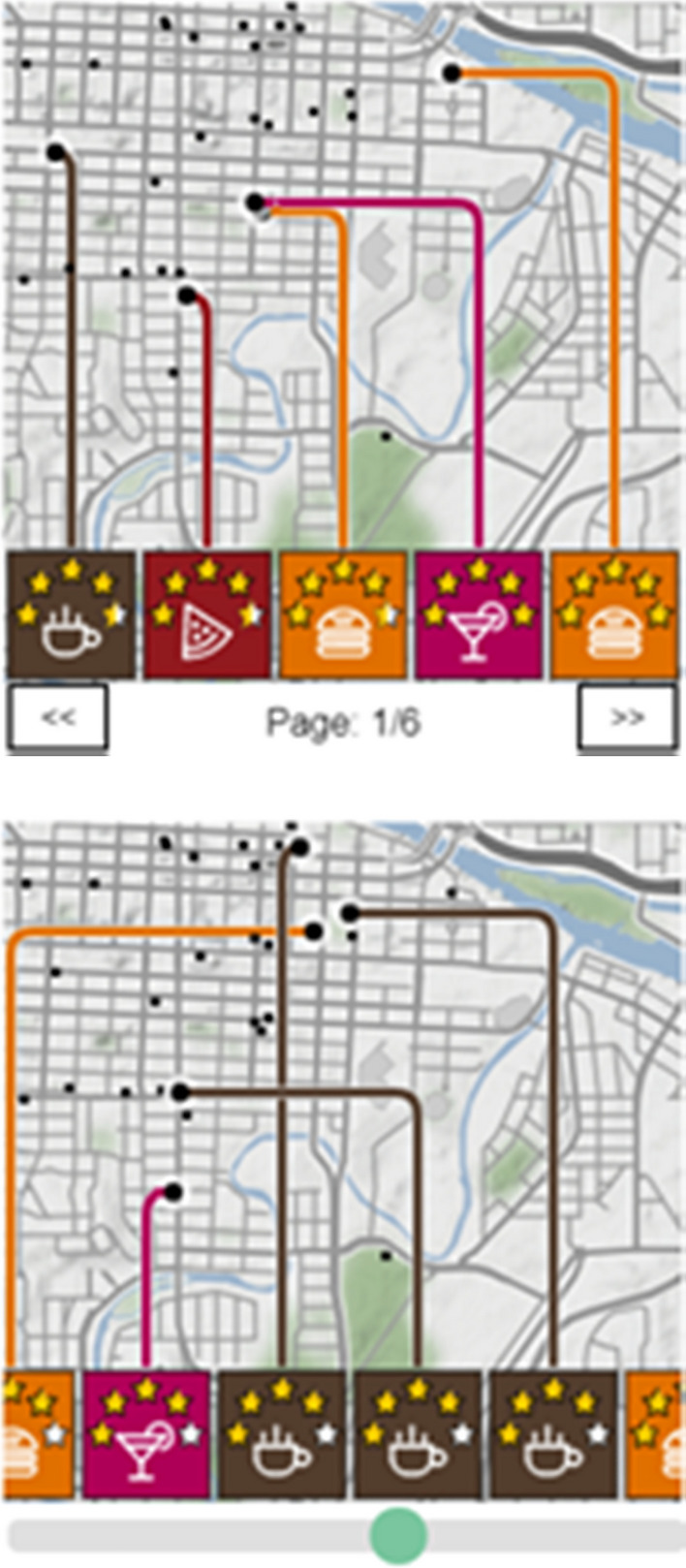
Two labeling models allow a user to access labels for all points on a map without zooming into a very large scale. Top: A model allowing a user to navigate from page to page. Bottom: A model based on a slider that can be used to let labels enter or leave the map (Image taken from a mobile application, leading to a reduced resolution)

Research on cartographic label placement has a relatively long tradition in computer science, where it is often considered as a packing problem asking to place as many labels as possible on the map without creating overlaps. However, the optimal solutions generated with this approach often result in a map full of labels, covering the features displayed in the map almost completely. Moreover, research on cartographic label placement has so far primarily dealt with static maps or with maps where zooming is the only possible interaction. Consequently, the existing solutions often require the user to zoom into an extremely large scale to receive a map in which all features are labeled. This is unsatisfactory especially for maps on small devices such as smartphones or smartwatches, where at a large scale only a small area can be shown, causing the user to lose the context. New labeling models and labeling algorithms for small-screen maps need to be developed to allow a user to access all labels at a medium scale, where a sufficient overview is still provided. An approach by Gedicke et al. (2020) is to use external labels, avoiding the occlusion of the background map and to allow the user to navigate through multiple views of the same map to access all labels (see Fig. 22). Efficient heuristic optimization methods are used to compute solutions in real-time, yet for the evaluation of the mathematical model that formalizes the labeling problem, exact optimization methods based on mathematical programming are of advantage. As this research shows, the combination of algorithm engineering and cartography has a large potential, which has not been fully exploited yet.

### Research Perspectives

**Fig. 23:**
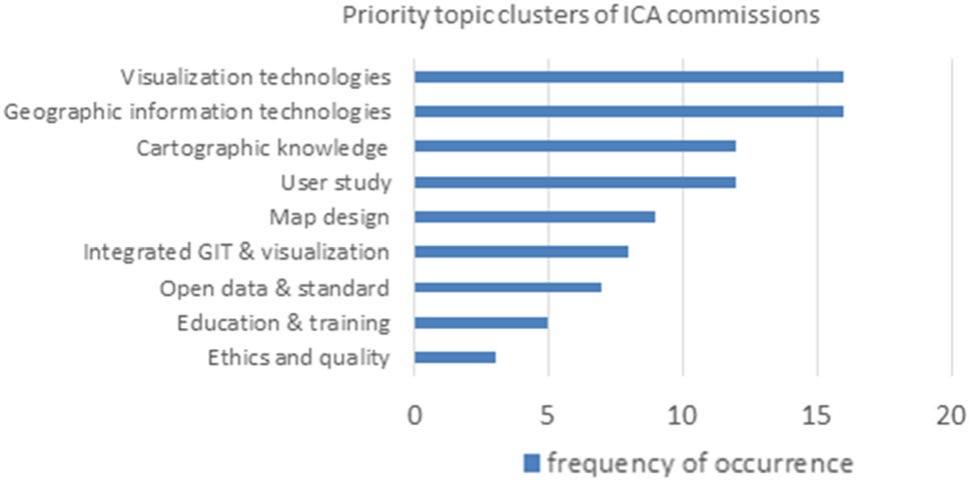
Research priorities of ICA commissions

**Fig. 24:**
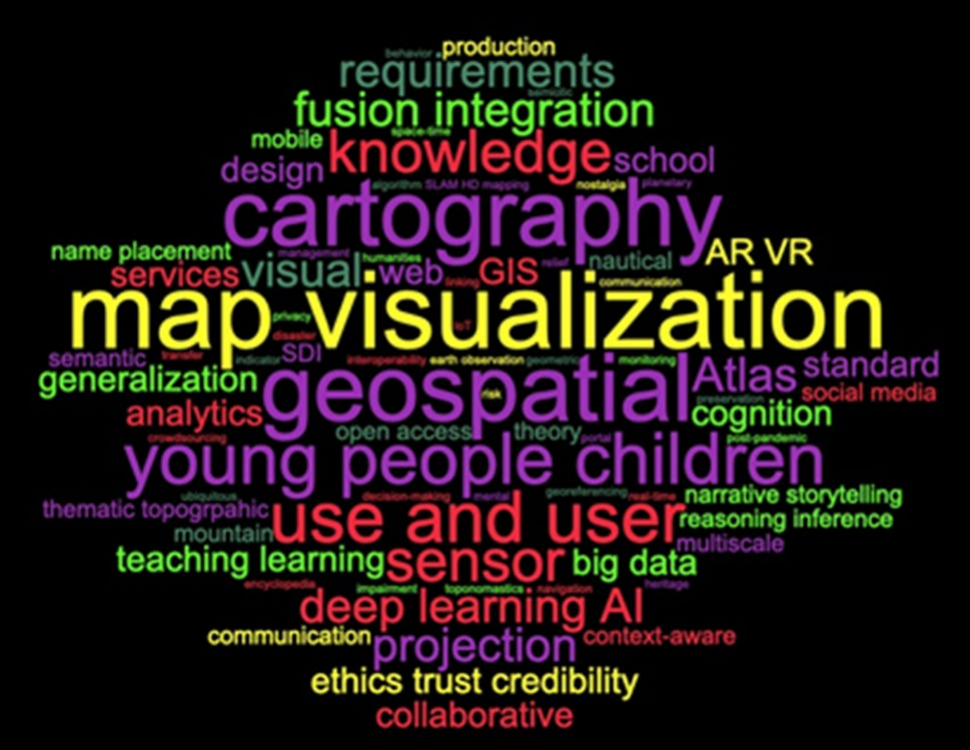
Frequently occurring keywords in priority topics

The aforementioned research works of DGK members are also being explored in the international community. A recent survey on the research priorities among 27 commissions of the International Cartographic Association (ICA) has revealed 9 topic clusters as shown in Fig. 23. “The two dominating clusters, i.e. visualization technologies and geographic information technologies, can very well justify the ICA as an international community for the promotion of the discipline and profession of cartography and GI science. The two other clusters, i.e. cartographic knowledge, including its preservation, accessibility and communication, and user study with research questions related to user experience, usability testing, human–computer interface etc., have also gained significant visibility. Integrated geographic information technologies and visualization has been a sustainable cluster for more than ten years and is characterized by topics of visual analytics. Open data and standards are a cluster with two mutually enhancing concepts. It has grown rapidly in recent years with open source platforms. Education and training covers both regular cartographic education programs for young people and life-long learning for mid-career professionals. Finally, there is an increasing awareness of ethics and quality issues in the value chain from raw data to user behavior, for which the role of cartographers is becoming indispensable.” (Meng et al. 2020). More detailed keywords and their frequencies of occurrence are represented in the word cloud in Fig. 24.

Concerning the automatic generation of maps, which falls into the cluster visualization technologies, algorithms and data structures are needed that can efficiently deal with large data sets. While in principle this challenge is not new, future maps will be more interactive, allowing the users to customize a map with multiple thematic, spatial, and temporal filters. Hence, an important task is to design multi-dimensional data structures which, after a set-up phase, can be queried in real time for a map corresponding to the filter settings specified by the user. These data structures need to be dynamic since new data are often incrementally added or even come in the form of continuous data streams.

A query, in this context, can be considered as a point in a multi-dimensional space. Sequences of queries issued by a user can be considered as trajectories in that space; e.g., a trajectory could represent the continuous change of the map scale during zooming or the interaction of a user with a time-slider interface. This idea also opens up new possibilities to study how users interact with maps. A particularly promising approach is to adapt methods of trajectory mining to analyze sequences of interactions. For example, using algorithms for trajectory clustering, one could identify groups of users with similar behaviors and preferences.

Another open challenge in the context of user studies is to define realistic tasks that can be quantitatively evaluated. There is a large agreement in the cartography community that a map should be tailored to a task, which is why task-based user studies are very popular and relevant. However, the tasks in such studies are often rather narrow, e.g., the participants are asked to find specified text labels on the map. Tasks that are more complex, such as tasks requiring a good overview for strategic planning, are rarely defined in such studies as they are more difficult to define and their solutions more difficult to assess.

Another promising direction of research is the development of new measures for the assessment of maps. In particular, measures of similarity for geometric objects are needed, e.g., to compare an automatically generated map with a reference solution or to assess the difference between two maps of different scales. Moreover, measures of visual complexity are needed to find a good trade-off between information fidelity and map legibility when generating a map from a large and detailed data set.

Finally, it is evident that in the future the links between the two disciplines cartography and information visualization will become even stronger. Algorithmic approaches to visualizing non-spatial and spatial (geographic) data are in principle the same, i.e., the same optimization techniques are applied in both fields. Moreover, the map is increasingly used as a metaphor for the visualization of non-spatial information, such as graphs or sets systems. For example, methods for visualizing author networks or instituational hierarchies as maps have recently found the interest of the InfoVis community. With their expertise in maps, cartographers have a lot to contribute to this area.

## Geospatial information dissemination

In recent years, the amount of geospatial information and the frequency of data acquisition (“velocity” in big data terms) have increased exponentially. Mainly, this change has come about by innovations in data collection such as novel surveying techniques and mobile mapping systems, geosensor networks, and remote sensing systems (e.g., Copernicus). Complementary to these developments the depth of penetration of the Internet in everyday life, as well as the progression of concepts such as Web 2.0–4.0 and crowdsourcing and crowdsensing, has increased significantly. The worldwide diffusion of small innovative sensors and smart devices created a variety of new endpoints apart from traditional computers. In the coming years this expansion of endpoints to the internet will continue and new edges, which are hardly foreseeable today, will emerge. Connectivity and digitalization are the main drivers of this development. With new wireless communication infrastructures, for instance, 5G, LoRaWAN, or NBIoT, but in the long-term also with initiatives such as StarLink, internet connectivity will propagate also to remote regions. At the same time, digitalization will continue to penetrate every aspect and object of human life. The advancement of embedded systems will continue to be characterized by miniaturization, but also energy autonomy with new concepts of energy harvesting will be pursued. The IoT will potentially connect all objects to one another, with or without human interaction. These new devices will link the physical with the digital world and observe and drive real-world phenomena. Since every object in the physical world has geospatial properties such as location, the amount and velocity of geospatial data will increase further.

### State of the Art

#### Geoinformation Dissemination Approaches

Dissemination of data on the internet, especially with the evolution of the web, is usually based on the request/response communication pattern. In the traditional web, users make requests to a server to retrieve or create information about a resource such as a website. In the geospatial world, this paradigm resulted in geo web services and Web APIs to retrieve geospatial data using protocols of the web. For instance, the INSPIRE initiative (European Commission 2021) uses different models and standards of the Open Geospatial Consortium to make geospatial data accessible. The INSPIRE directive specifies a framework for the member states of the European Union to establish and operate local, regional and national spatial data infrastructures (SDIs) (Minghini et al. 2020). Thereby it ensures compatibility and usability across borders. Currently, it focuses on retrieving data with portrayal and data services defined by the OGC. WMS and WFS are two widely used services to request maps or geospatial objects from servers (Beaujardiere 2006; Vretanos 2010). With the introduction of the Sensor Web Enablement (SWE) services such as Sensor Observation Service (SOS) or the SensorThings API, these infrastructures are extended to sensor observations and tasking in the IoT. With the standardization of the SensorThings API (Liang et al. 2016) and recently the OGC API – Features (Portele et al. 2019), the service-based focus of the OGC has shifted towards the Web API approach. The design pattern allows a user and developer-friendly easy-to-use interface based on common web technologies such as HTML or GeoJSON standardized (geo) services, Web APIs, and communication protocols to provide interoperability between (spatial) data infrastructures and enhances information fusion, for instance, in web applications such as geoportals.

#### Message-Driven Communication

However, nowadays, many real-world applications are reactive systems, which are based on asynchronous message-driven interaction models. They often rely on loosely coupled push messaging, which is initiated by the publisher and not by the requestor. Hereby, clients express their information preferences in advance, for instance with a publish/subscribe model. With this messaging mechanism, the continuous real-time delivery of data tuples between producer and consumer is possible, so that also data streams can be initiated. Rieke et al. (2018) identify the need for message-driven models in contemporary SDIs to support the exchange of geospatial information in real-time. In recent years, some efforts from the OGC to establish push-based dissemination standards of geospatial data have already been made. Initially driven by the sensor web community, these have resulted in the OGC publish/subscribe interface standard, which includes metadata, but relies on other message delivery protocols such as SOAP (Simple Object Access Protocol) messaging or AMQP (Advanced Message Queuing Protocol). By now, there is no designated push-based geospatial standard which disseminates spatially annotated messages based on push technology. Such a standard might help to disseminate messages based on geospatial properties. However, some research has been conducted in this field recently (e.g., Hasenburg and Bermbach 2020; Herlé 2019).

### Case Studies

#### Case Study “EarlyDike—An Early Warning System for Dike Failures”

**Fig. 25:**
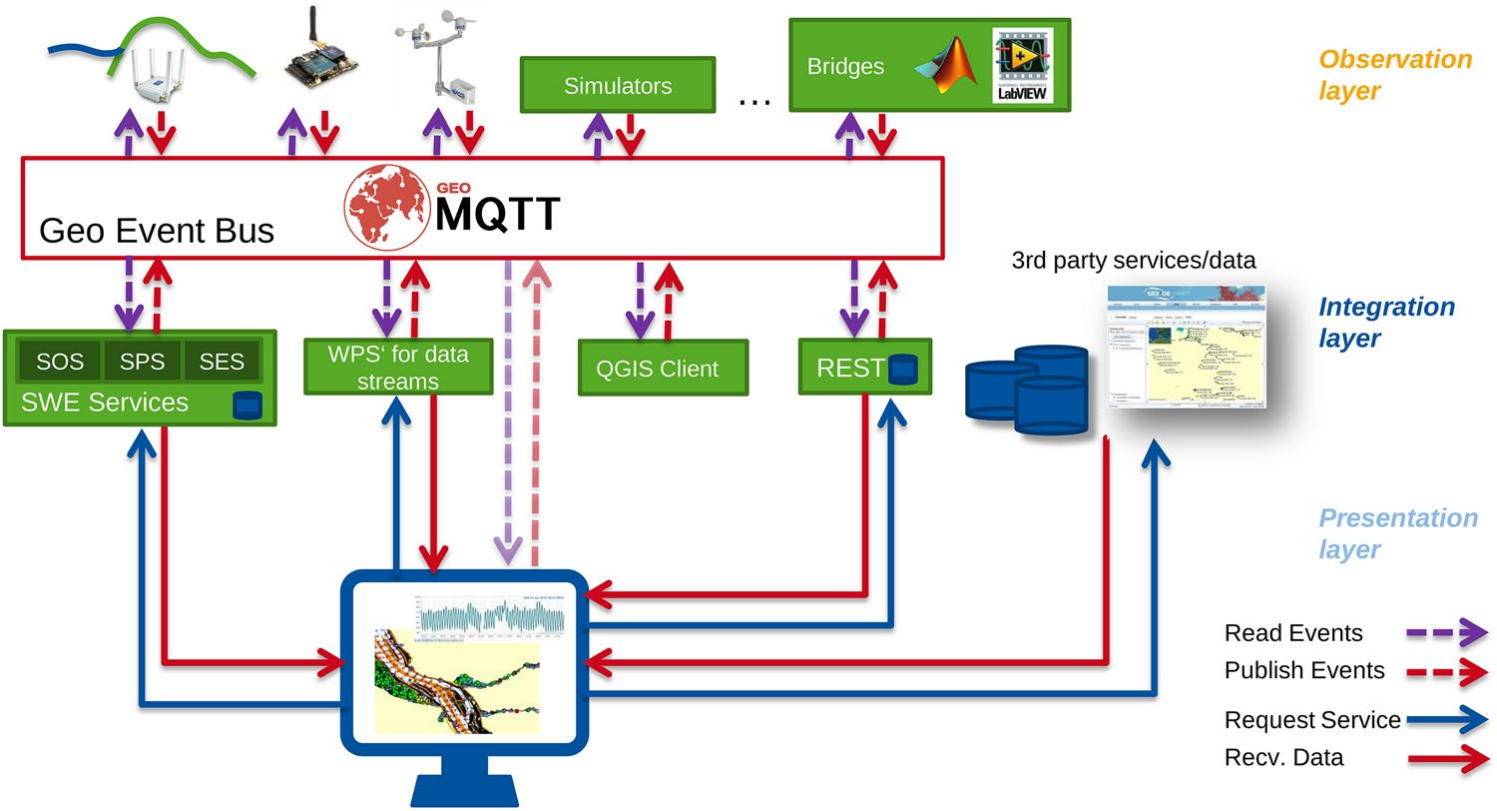
Architecture with GeoEvent Bus in the EarlyDike Project (adapted from Herlé et al. 2016)

In the EarlyDike project, an early warning system for dike failures based on real-time geospatial events is realized (Becker et al. 2016; Herlé et al. 2016). The project consists of three methodological approaches: (i) simulation of the environmental impacts by forecasting storm surges and wave loads, (ii) monitoring of the condition of the dike itself by deploying new sensor technologies, and (iii) simulation of the extent of a flood in case of a dike failure. The architecture of the projects consists of three layers (see Fig. 25). The backend forms a sensor and spatial data infrastructure (SSDI) with an observation and an integration layer. Services of the SWE such as the SOS are used to store and retrieve the data produced in the project. These can be requested by the presentation layer, e.g., a Geo Portal, by utilizing a request/response pattern. Additionally, the eventing between the software parts is introduced by IoT protocols. The GeoMQTT protocol is used to establish a geo event bus to interconnect sensors, simulators, and the SSDI (Herlé 2019). It ensures the delivery and provisioning of real-time spatio-temporal events and data in time-sensitive architectures such as early warning systems.

#### Case Study “senseBox and openSenseMap”

**Fig. 26:**
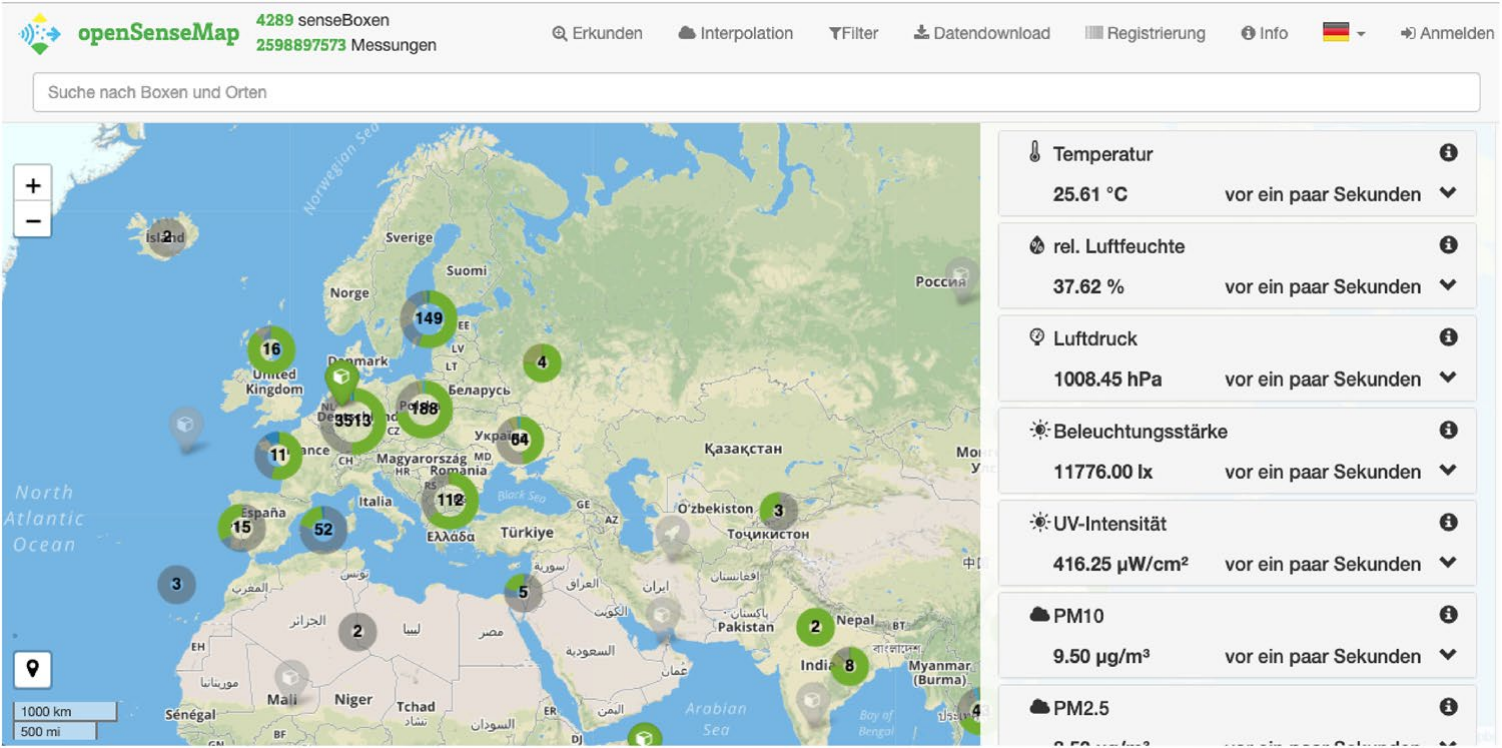
The openSenseMap is a citizen science web platform for environmental data (Pesch and Bartoschek 2019)

The senseBox is an open-source Do It Yourself (DIY) citizen science toolkit which can be used to monitor the environment (Bartoschek et al. 2018). This crowdsensing approach addresses citizens and especially high school students to participate in an open monitoring network for various environmental phenomena such as temperature, particulates, or noise. The senseBox can be easily connected to the openSenseMap (https://opensensemap.org/), a sensor web platform to store, analyze and provide continuous measurement data (see Fig. 26).

The web platform provides open interfaces, so that users from other citizen science projects such as luftdaten.info (https://luftdaten.info/) or hackAIR (https://platform.hackair.eu/) may use openSenseMap as a platform for storing and analyzing their data.

### Research Perspectives

#### Technology Enables Novel Distributed Geo Applications

Novel applications in different domains will appear that make use of new internet edges and their geospatial events. These include environmental monitoring and structural health monitoring (SHM) in smart cities or smart buildings or, in general, digital twin applications. Also, moving objects and associated applications will benefit from increased connectivity and digitalization. In transportation and logistics, new concepts based on inter-object communication and geospatial data streams will be implemented to find solutions for current traffic problems. Vehicle to vehicle (V2V), vehicle to infrastructure (V2I), and the Internet of UAVs (IoU-AV) are just some of the promising concepts, which can be implemented by the innovative possibilities this technology offers. In addition, applications of different domains such as the Internet of Production (IoP) or the Internet of Construction (IoC) will use geospatial data, so that the dissemination is crucial for different use cases. However, the dissemination of geospatial data raises a multitude of research questions and challenges in the future which have to be tackled to make it possible to implement applications and increase efficiency as well as sustainability.

#### Future Data Infrastructures

Future geospatial data infrastructures follow various goals and face different challenges: from improving productivity and efficiency over engaging citizens as data providers and users to ensuring individual privacy and tracking of the provenance of data (Dangermond and Goodchild 2020). For instance, in the future, the SDI approach manifested, e.g., in the INSPIRE directive could be extended to processing or sensor services. Also, advanced querying languages such as GraphQL and similar technologies should be considered in these architectures (Shelden et al. 2020). Additionally, the interoperability focus should not be exclusively on the geospatial information modeling world. New promising related concepts and data collecting approaches foster the integration of different domain applications and data sources. The IoT or other closely related domains such as BIM share common geospatial objects and should be considered in interoperability approaches. Thus, interoperability strategies across domains need to be developed and established (Herlé et al. 2020). Since current infrastructures mainly focus on the request/response principle, the already mentioned push technologies which promote real-time geospatial event streams need to be considered in next-generation infrastructures and architectures as well. This integration and the vast amount of events create various research questions and challenges concerning especially the selection and aggregation of these data streams.

#### Finding Meaningful Messages and Events

Systems that communicate by messages or events need to be able to distinguish between meaningful and meaningless messages. Based on e.g. context, location, proximity or semantics, messages have to be filtered while disseminated to address the correct recipients. In systems with large numbers of publishers, these filtering processes have to be applied to potentially a high number of messages with high velocity. This requires real-time analytics and the fusion of data streams to filter meaningful messages. Extraction and dissemination of meaningful events become even more complex when information from different domains and systems are fused. On the one hand, new opportunities arise; on the other hand, interoperability challenges increase further. Automatic curation, transformation, and fusion of events and data pose new research questions. Technologies of the semantic web such as linked data structures and ontologies, but also artificial intelligence and machine learning can be one solution to successfully deliver meaningful messages to the correct recipients. Besides typical security issues such as authentication or privacy, open challenges include questions about the provenance and validity of messages and encapsulated data. Especially in forecast and decision support systems suitable extensions and mechanisms are needed to model uncertainty and quality of the data. New mechanisms and extensions also have to be designed to meet interoperability requirements and the nature of geospatial data.

#### National Research Data Infrastructures

Furthermore, big geospatial data with high volume and velocity such as remote sensing data gathered by Copernicus or from geosensor networks introduce new types of data that currently cannot be integrated into SDIs using the existing mechanisms and standards. Usually, depending on the type and the use case, dedicated data infrastructures are conceptualized and implemented. These dedicated data infrastructures often form data silos which are typically difficult to access, especially if proprietary protocols and formats are used. Therefore, SDIs must be extended to adopt future requirements. For instance, in Germany, several research communities are launching national data infrastructures for different kind of scientific data (Nationale Forschungsdateninfrastruktur, NFDI, see Sect. 9).

## Framework for GI Research in Germany

### Infrastructures

The directive *“*Infrastructure* for Spatial Information in Europe (INSPIRE)”* develops a framework for the member states of the European Union to establish and operate local, regional, and national SDIs. Thereby, it ensures compatibility and usability across borders. Currently, it focuses on retrieving data with portrayal and data services defined by the OGC. In the future, the approach could be extended to processing or sensor services. INSPIRE can be seen as state of the art in geospatial data management.

The directive on open data and the re-use of public sector information (‘Open Data Directive’, EU 2019/1024, http://data.europa.eu/eli/dir/2019/1024/oj) provides common rules for a European market for government-held open data (https://digital-strategy.ec.europa.eu/en/policies/open-data). This will stimulate the publishing of dynamic data and the uptake of Application Programming Interfaces (APIs) and limit the exceptions which currently allow public bodies to charge more than the marginal costs of dissemination for the re-use of their data. *Open Data* will become the default in the future, which will support much more data-driven geospatial information research and usage.

DFG RISources (https://risources.dfg.de/) is an information portal on *scientific research infrastructures* in Germany that provides scientists with resources and services for planning and carrying out research projects. Some examples of already existing infrastructure components are open access repositories (iDAI.geoserver, BonaRes, Coastal Observing System for Northern and Arctic Seas (COSYNA), GLUES Geodata Infrastructure (GLUES GDI), Live weather data from German sites (WETTER), Animal Tracking Data (Movebank) and virtual cultural landscape laboratories (Virtuelles Kulturlandschaftslaboratorium (VKLandLab, Bill 2012)).

There is a growing awareness in the research community of the importance of *FAIR principles* in data handling: data should be free, accessible, interoperable, and reusable (Wilkinson et al. 2016). This implies the necessity of structured data storage with automatic search mechanisms on rich metadata. Many countries in recent years launched calls to set up national research data infrastructure (e.g. in Germany by DFG https://www.dfg.de/foerderung/programme/nfdi/), intended to systematically develop, sustainably secure, and make accessible the data holdings of science and research and to network them (inter)nationally. They will be established in a process driven by the scientific community as a networked structure of consortia acting on their initiative.

With *NFDI4Earth* (https://www.nfdi4earth.de/) the german earth system science community is developing such an infrastructure based on the FAIR principles. The concept includes several standards from the geospatial world to ensure interoperability and sustainability. Especially huge amounts of spatio-temporal data must be exploited and combined, which will raise new enormous challenges on existing or future mechanisms and standards. Despite the efforts for interoperability, the seamless interconnectivity with existing SDIs is not given, however, desirable. Mechanisms for linking data sets across boundaries of data infrastructures of different domains in an interoperable way have to be investigated. Solving this challenge has huge potential for increasing efficiency and forming new applications.

In summary, the public administration supports science and economy by providing up-to-date and adapted geodata and services. The expansion of spatial data infrastructures in the administration (such as INSPIRE) and economy is well advanced. And in science, too, the development and networking of data repositories from different disciplines are being driven forward, e.g. through the NFDI initiative. Still, the geoinformatics community seems to rely on existing infrastructure for their research, not (yet) seeing the necessity of jointly pushing forward the establishment of a joint infrastructure, serving several purposes in an integrated way, e.g. an urban observatory (building, energy, pollutants, waste, social aspects), or a mobility observatory (individual mobility, logistics, traffic control). Beyond physical infrastructures and data infrastructures there is also an increasing need for software infrastructures, which allow to exploit the richness of the data in open and reproducible ways.

### Research Funding

Funding of research is as diverse and differentiated as the research landscape itself. Different national and European research funding agencies at different levels and with different intentions exist: From a german perspective, these are the EU (Horizon 2020), DFG with more basic research funding, BMBF (e.g. FONA), BMWK (e.g. Copernicus related research), and BMVI (e.g. MFUND), which are more dedicated to applied research. Three large coordinated research projects are described in the following.

DFG currently funds a priority program (“Volunteered Geographic Information: Interpretation, Visualizierung und Social Computing” (SPP 1894)) coordinated by TU Dresden. “The core question of this SPP is how visualization methods can contribute to the utilization of VGI and support the interaction of users involved. Research questions span from the extraction of spatial information to the visual analysis and knowledge presentation, taking into account the social context while collecting and using VGI. The heterogeneity and the limited semantic structure of user-generated spatial data represent a major challenge.” (https://www.vgiscience.org/).

Also the research training group RTG 2159 on “Integrity and Collaboration in Dynamic Sensor Networks” (https://www.icsens.uni-hannover.de/en/), a joint doctoral program at Leibniz University Hannover, is funded by DFG. Collaborative sensor networks which need to guarantee integrity are a core element of many future systems. Examples are automated and autonomous vehicles, but such systems are also found in flexible factory automation, agriculture, construction, service and home robotics. In particular, multi-sensor platforms moving around in the real world and communicating with each other must ensure the integrity of their navigation information, in order not to endanger their environment. The prime contribution of the RTG is to provide basic methodologies as well as concepts of integrity and collaboration for dynamic sensor networks in connection with digital maps (Schön et al. 2018).

One of the significant challenges facing our society concerning agriculture is to increase crop production, despite limited arable land, and at the same time, reduce the ecological impact. The DFG-funded cluster of excellence “Phenorob: Robotics and Phenotyping for Sustainable Crop Production” (https://www.phenorob.de/) at the University of Bonn is developing methods and new technologies that observe, analyze, better understand and specifically treat plants. Different sensor platforms, i.e., ground and aerial vehicles, operated autonomously, provide precisely georeferenced and phenotypic data from single plants over the experimental plot to the field scale. 3D structural models of one and the same plant will be registered over time for a 4D reconstruction. This will lead to the development of a new generation of mapping systems and a better understanding of the spatio-temporal dynamics of structural and functional plant traits. The project develops an autonomous field robot that detects and identifies individual plants and creates a weed map of the field to treat individual plants with the most appropriate intervention method. Furthermore, the robot can precisely apply nitrogen fertilizer enabled by digital avatars that predict the plant nutrient demand and probable losses in the field.

## Geospatial Information Research and the Grand Challenges

### Grand Challenges

Grand challenges are medium to long-term focused topics that require significant breakthroughs in basic research, applied research, and technical development in key technologies. One of their primary objectives is to focus and align research efforts: Scenarios are developed that should be easy to communicate and understand. The goal must be very ambitious and be at the limit of what is "just about possible". At the same time, there should be hope that these goals can realistically be achieved (Mertens and Barbian 2015; Wissenschaftsrat 2015). There exist different lists of grand challenges; most of them include health, demographic change, and wellbeing; food security, sustainable agriculture, and forestry; marine and maritime and inland water research, and the bioeconomy; secure, clean, and efficient energy; smart, green and integrated transport; climate action, environment, resource efficiency, and raw materials; changing world, inclusive, innovative and reflective societies; secure societies – protecting freedom and security worldwide.

In a similar vein, the United Nations, in 2015, have identified 17 sustainable development goals addressing the most pressing problems facing our world (https://sdgs.un.org/goals, Kraak et al. 2021).

As a result of scientific and technical progress, for example in laser, information, and satellite technology, the core tasks in geoinformatics can be performed ever faster and more cost-effectively, but above all more comprehensively, with far higher quality and rapidly increasing spatial and temporal resolution. In recent years, the all-encompassing digital transformation has entered science far more than before by opening up new ways of thinking and enabling new methods. In the course of the digital transformation, there is a growing realization in many disciplines that a uniform, high-quality reference to space and time in conjunction with digital environmental information of all kinds is imperative as an ordering scheme for many questions. The references to our living space and the systemic understanding that has grown in the process offer increased points of contact for geospatial information processing with societal mega topics, such as mobility, information, energy, climate, health, and environment.

### GI community Contributions

The geospatial information science community can contribute to the solution of nearly all grand challenges, with its expertise in geospatial data acquisition and interpretation, information modeling and management, information integration, decision support, information visualization, and information dissemination. Geoinformatics provides methods for the collection and interpretation of sensor data for the extraction of geoinformation as well as for its management, analysis, and visualization. It thus provides the basis for the generation of digital representations of the real world (digital twin) being of essential importance for tackling major societal challenges. For example, geoinformatics contributes to more sustainable agriculture and the development of new mobility concepts. The development of methods increasingly relates to machine learning procedures. In addition, new research topics are emerging, e.g. at the interface between humans and autonomous system: enhancing and supporting human actions and human understanding by increasing assistance and semantics through adequate interaction interfaces, to name only a few.

**Fig. 27:**
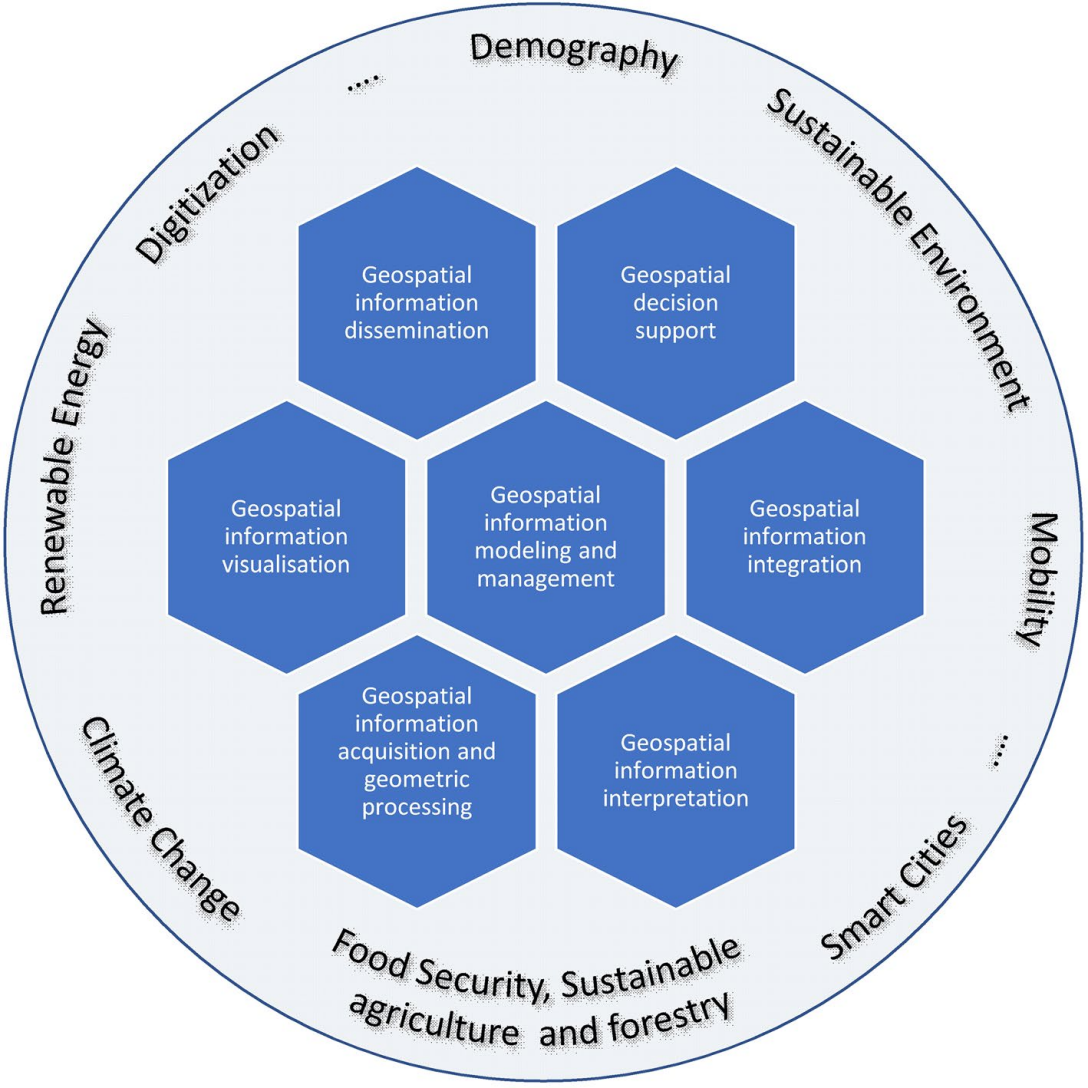
GI research contributions to the grand challenges

Those challenges are beyond what a single discipline can handle, thus, this calls for an increasing interdisciplinary collaboration with other disciplines disciplines—and also with society as a whole. Only recently, the National Academy of Sciences Leopoldina has called for such a collaboration to establish and foster a *Earth System Science* in Germany (German National Academy of Sciences Leopoldina 2022). As spatial data and their processing are core, the GI community should not be too shy to also take a leading role (Fig. 27).

## Data Availability

All URLs are checked end of March 2022.

## Abbreviations

4As: Anytime, anywhere, for anyone, and anything
AAN: Appearance adaptation network
AI: Artificial intelligence
ALS: Airborne laser scanning
AMQP: Advanced message queuing protocol
API: Application programming interfaces
AR: Augmented reality
ATKIS: Amtliches Topographisch-Kartographisches Informations system
BIM: Building information model
B-Rep: Boundary representations
CAD: Computer-aided design
CNN: Convolutional neural networks
CP: Computational photography
CRF: Conditional random fields
CSG: Constructed solid geometry
DE: Desktop environment
DFG: Deutsche Forschungsgemeinschaft
DGK: Deutsche Geodätische Kommission
DIY: Do it yourself
DL: Deep learning
DSMS: Data stream management systems
EO: Earth observation
ESA: European space agency
GAN: Generative adversarial networks
GC: Grand challenges
GDF: Geographic data files
GI: Geospatial information
GIM: Geospatial information modeling
GIS: Geographic information system
GML: Geography markup language
GNSS: Global navigation satellite systems
GSD: Ground sampling distance
HD: High definition
ICA: International cartographic association
ICT: Information and communication technologies
IMU: Inertial measuring unit
IFC: Industry foundation class
INS: Inertial navigation system
InSAR: Interferometric synthetic aperture radar
INSPIRE: Infrastructure for spatial information in Europe
IoC: Internet-of-construction
IoP: Internet-of-production
IoT: Internet of things
IoUAV: Internet of UAVs
ISO: International Organization of Standardization
ISPRS: International Society for Photogrammetry and Remote Sensing
LADM: Land administration domain model
LandInfra: Land and infrastructure conceptual model
LBS: Location-based services
LCZ: Local climate zones
LoD: Level of detail
LOD: Linked open data
LSTM: Long short-term memory
MCDA: Multi criteria decision analysis
MCMC: Markov Chain Monte Carlo
MR: Mixed reality
MRF: Markov random fields
NASA: National aeronautics and space administration
NFDI: Nationale Forschungsdateninfrastruktur
OGC: Open geospatial consortium
OODBMS: Object-oriented database management systems
ORDBMS: Object-relational database management systems
POI: Point-of-interest
PSI: Public sector information
RADAR: RAdio detection and ranging
RANSAC: Random sample consensus
RDBMS: Relational database management systems
RDF: Resource description framework
RNN: Recurrent neural networks
SDBMS: Spatial database management systems
SDG: Sustainable development goals
SDI: Spatial data infrastructures
SDSS: Spatial decision support system
SHM: Structural health monitoring
SOAP: Simple object access protocol
SOS: Sensor observation service
SQL: Structured query language
SRS: Spatial reference system
SSDI: Sensor and spatial data infrastructure
STDBMS: Spatio-temporal database management systems
SWE: Sensor web enablement
UAV: Unmanned aerial vehicles
UML: Unified modelling language
UN: United Nations
V2I: Vehicle-to-infrastructure
V2V: Vehicle-to-vehicle
VGI: Volunteered geographic information
VR: Virtual reality
WMS: Web map service
WFS: Web feature service
WPS: Web processing service
XML: Extensible markup language

## Code Availability

Not relevant.
